# Predicting the progression of MCI and Alzheimer’s disease on structural brain integrity and other features with machine learning

**DOI:** 10.1007/s11357-025-01626-5

**Published:** 2025-04-26

**Authors:** Marthe Mieling, Mushfa Yousuf, Nico Bunzeck

**Affiliations:** 1 Department of Psychology, University of Lübeck, Ratzeburger Allee 160, 23562 Lübeck, Germany; 2 Center of Brain, Behavior and Metabolism (CBBM), University of Lübeck, Ratzeburger Allee 160, 23562 Lübeck, Germany

**Keywords:** Alzheimer’s disease, Magnetic resonance imaging, Structural degeneration, Machine learning, Classification

## Abstract

Machine learning (ML) on structural MRI data shows high potential for classifying Alzheimer’s disease (AD) progression, but the specific contribution of brain regions, demographics, and proteinopathy remains unclear. Using Alzheimer’s Disease Neuroimaging Initiative (ADNI) data, we applied an extreme gradient-boosting algorithm and SHAP (SHapley Additive exPlanations) values to classify cognitively normal (CN) older adults, those with mild cognitive impairment (MCI) and AD dementia patients. Features included structural MRI, CSF status, demographics, and genetic data. Analyses comprised one cross-sectional multi-class classification (CN vs. MCI vs. AD dementia, *n* = 568) and two longitudinal binary-class classifications (CN-to-MCI converters vs. CN stable, *n* = 92; MCI-to-AD converters vs. MCI stable, *n* = 378). All classifications achieved 70–77% accuracy and 61–83% precision. Key features were CSF status, hippocampal volume, entorhinal thickness, and amygdala volume, with a clear dissociation: hippocampal properties contributed to the conversion to MCI, while the entorhinal cortex characterized the conversion to AD dementia. The findings highlight explainable, trajectory-specific insights into AD progression.

**Supplementary Information:**

The online version contains supplementary material available at 10.1007/s11357-025-01626-5.

## Introduction

Structural brain integrity has long been associated with cognitive abilities across the adult lifespan [1–3]. While a distinction can be made between healthy vs. pathological changes in mild cognitive impairment (MCI) and Alzheimer’s disease (AD) dementia, it is also clear that age-related changes can be described on a continuum rather than one-off events, and, therefore, understanding developmental trajectories is essential. In this regard, machine learning [4] offers novel ways to systematically investigate the contribution and developmental changes of complex feature constellations, including brain regions, proteinopathy, or genetic factors; however, longitudinal analyses using machine learning with cognitively normal (CN) older adults, MCI, and AD dementia patients are scares. Filling this apparent gap, and identifying fingerprints of complex feature constellations in the context of AD progression, is the goal of this work.

Apart from typical neurodegeneration, especially within medial temporal lobe regions, such as the hippocampus and entorhinal cortex, the progression of AD is associated with the accumulation of amyloid-β (Aβ) and hyperphosphorylated tau (pTau) [5–7]. These neuropathologies may all serve as biomarkers, but the initial stages of AD often manifest without noticeable symptoms, making it challenging to identify them early on [7]. Previous research indicates that the progression of AD is distinguished by the dissemination of structural brain degeneration, which is influenced by Aβ and pTau proteinopathy, quantified based on cerebrospinal fluid (CSF) assays. Accordingly, initial structural degeneration spreads from subcortical brain regions, such as the nucleus basalis of Meynert (NbM) but also possibly the locus coeruleus [8], to the entorhinal cortex moderated by CSF status [9, 10]. Importantly, this is followed by a spread of neural degeneration from the entorhinal cortex to temporal and parietal brain regions, which can be detected in vivo by structural MRI [9] even in preclinical AD. For instance, subjects with preclinical AD (i.e., cognitively normal and abnormal Aβ and pTau biomarkers) showed more atrophy in the entorhinal cortex than cognitively healthy (i.e., cognitively normal, normal Aβ, and pTau biomarkers) [9]. Hence, the development of AD can be described on a continuum encompassing normal aging, initial pathological shifts in biomarkers, followed by the onset of mild symptoms as seen in MCI, finally leading to the development of AD dementia [7]. In this regard, CSF status and MRI measures of structural brain integrity appear to be important biomarkers [5, 11].

Previous studies have already used different machine learning approaches to classify high-performing CN, MCI, and AD dementia patients based on structural MR images [12–14]. Classification accuracies in binary classifications ranged from 80 to 100% for CN vs AD dementia, 60–90% for CN vs. MCI, and 50–85% for AD dementia vs MCI [15, 16]. These effects were often based on lower volumes in MCI and AD dementia as compared to CN in specific brain regions, which is in line with the prevailing view on the etiological and neuroscientific comprehension of AD dementia [17–21]. These include the hippocampus, amygdala, entorhinal cortex, precuneus, cingulate gyrus, and the rostral and caudal regions within the medial frontal lobe [22]. In particular, hippocampal and entorhinal integrity plays a crucial role in the progression and prediction of AD [13, 23, 24] since atrophy in these regions strongly predicts future cognitive impairment and serves as an early indicator of AD dementia [25–28]. Despite the apparent specificity, especially within the medial temporal lobe, AD encompasses widespread neurodegenerative processes throughout the entire brain [17], which have not fully been addressed. Along these lines, research on ternary classifications of CN vs. MCI vs. AD dementia, and longitudinal analyses using machine learning on the disease progression, involving the conversion from CN to MCI or MCI to AD dementia, are scares or do not provide information on the contributing features (i.e., brain regions) of the classification [13–15, 29–31].

**Fig. 1:**
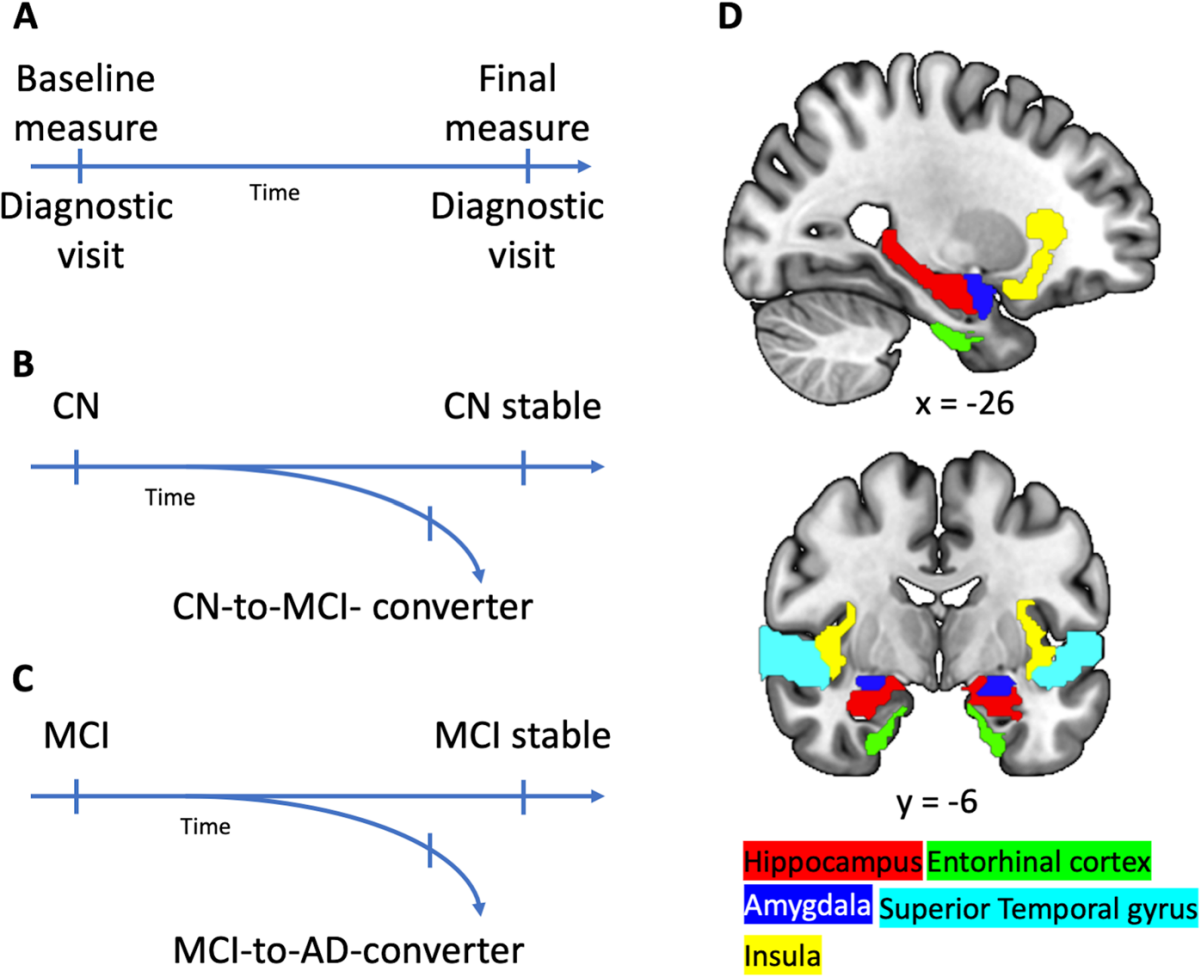
Schematic illustration of the experimental design and analyses. All subjects underwent at least two diagnostic visits, including a baseline measure and follow-up (**A**). In a first cross-sectional analysis, cognitively normal (CN) older adults were compared against patients with mild-cognitive impairment (MCI) and patients with Alzheimer’s disease dementia (AD). **B** depicts analysis 2 with CN that either remained stable over all follow-ups or developed a MCI at a following measure. **C** Analysis 3 with MCI patients who remained stable and MCI patients who later converted to AD dementia. **D** Illustration of those brain regions that, across analyses, emerged as particularly relevant. Regions, except for the entorhinal cortex, were extracted based on the Automated Anatomical Labeling (AAL) atlas [32], while the entorhinal cortex was extracted using the Anatomy Toolbox [33–35] and overlayed onto a T1-weighted standard brain template

Therefore, we utilized data from the Alzheimer’s Disease Neuroimaging Initiative (ADNI) to examine how differences in brain integrity (volume and thickness) and additional features, such as CSF status, age, sex, education, and APOE4 genotyping, contribute to the classification of CN vs. MCI vs. AD dementia (cross-sectional, analysis 1, Fig. 1A). In a second step, we performed a longitudinal binary-class classification of CN older adults who later converted to MCI vs those that remained healthy (i.e., CN-to-MCI converters vs. CN stable, analysis 2, Fig. 1B), and MCI patients that later converted to AD dementia vs those that remained MCI (i.e., MCI-to-AD converters vs. MCI stable, analysis 3, Fig. 1C). All three analyses were based on state-of-the-art XGBoost (eXtreme Gradient Boosting) machine learning algorithm, and the assessment of SHAP (SHapley Additive exPlanations) values to pinpoint the contributing features for the classification models. We expected that the integrity of medial temporal lobe brain regions, especially the hippocampus and entorhinal cortex, contribute to the distinction of CN, MCI, and AD dementia, and, importantly, they would allow a precise prediction of the disease progression.

## Methods

### ADNI data

Data used in the preparation of this article were obtained from the Alzheimer’s Disease Neuroimaging Initiative (ADNI) database (adni.loni.usc.edu). ADNI was launched in 2003 as a public–private partnership, led by Principal Investigator Michael W. Weiner, MD, to test whether serial magnetic resonance imaging (MRI), positron emission tomography (PET), other biological markers, and clinical and neuropsychological assessment can be combined to characterize the progression of mild cognitive impairment (MCI) and early Alzheimer’s disease.

### Participants and diagnoses

We included participants based on their diagnosis as CN, MCI, and AD dementia at the time point of MRI measurement. The assignment of diagnoses was conducted by the ADNI Clinical Core, which categorized individuals into three groups: CN (Clinical dementia rating [CDR] = 0, Mini Mental State Examination [MMSE] = 24–30), MCI (CDR = 0.5, MMSE = 24–30), and AD dementia (CDR = 0.5–1, MMSE = 20–26). These classifications are commonly used in clinical trials to assess cognitive and functional measures [36, 37] (for more information on the diagnostic procedure and in- and exclusion criteria, please see http://adni.loni.usc.edu).

For the first analysis (CN vs. MCI vs. AD dementia, Fig. 1A), the disease classification was based on a participant’s baseline measures. In the longitudinal analyses 2 (CN-to-MCI converters vs CN stable, Fig. 1B) and 3 (MCI-to-AD converters vs MCI stable, Fig. 1C), for the converters (CN-to-MCI converters and MCI-to-AD converters) the latest available measurement as CN before the conversion to MCI (minimum 4 months), and AD dementia (minimum 4 months), respectively, was selected. Therefore, the term “longitudinal” refers to the diagnostic information rather than changes in MRI measures. For CN stable and MCI stable, the earliest available measurement was chosen (e.g., baseline information or if MCI converted from CN, then the first measurement associated with MCI), and time of stability was assessed by the time interval between the earliest available measurement and the last available ADNI visit. These participants remained stable for at least one measurement or longer and did not undergo conversion in their follow-up visits recorded by ADNI. For all analyses, participants with reverting diagnoses (e.g., MCI to CN, or AD dementia to MCI) were excluded.

Participants were only included with available high-quality FreeSurfer MRI, CSF drawings, and genotyping data acquired in close proximity with their neuropsychological assessment (< 6 months ago) (for more specific information regarding the criteria for inclusion and exclusion in ADNI, see http://adni.loni.usc.edu). Table S1 gives an overview of the participants’ demographics, information on APOE4 genotype and harmonized CSF assay Table S2 shows neuropsychological test results.

### MRI acquisition and FreeSurfer processing

The participants’ MRI scans were obtained at various sites equipped with 1.5 and 3.0 Tesla scanners, following the standardized monitoring protocols of ADNI (see http://adni.loni.usc.edu for more information). Importantly, the available T1 images were already processed by ADNI (the Tosun laboratory at the University of California-San Francisco (see https://adni.loni.usc.edu/data-samples/data-types/mri/) using the cross-sectional pipeline [38] in FreeSurfer [39] (version 4.3, 5.1 and 6.0, https://surfer.nmr.mgh.harvard.edu/). FreeSurfer is an open-source software package for processing, analyzing, and visualizing brain images (https://surfer.nmr.mgh.harvard.edu/). The features were extracted based on the Desikan-Killany atlas [40] and the subcortical whole brain segmentation [41, 42]. The tables provided by ADNI (versions 4.3, 5.1, and 6.0) were compiled by us, and the corresponding participants were selected accordingly.

In ADNI 1/GO, FreeSurfer version 4.3 encompassed data acquired from 1.5 Tesla scanners, version 5.1 included data from 3.0 Tesla scanners in ADNI GO/2, and version 6.0 integrated data from 3.0 Tesla scanners in ADNI 3. We merged FreeSurfer results, recognizing a potential bias [43]; however, FreeSurfer morphometric techniques have displayed strong test–retest reliability across various scanner manufacturers and field strengths [44]. Due to a limited number of participants in analyses 2 and 3, we combined data from 1.5 Tesla and 3.0 Tesla scanners, including all available FreeSurfer versions. In analysis 1, which had a sufficient number of participants, we adopted a more stringent approach by only including data from 3.0 Tesla scanners, utilizing the newer FreeSurfer versions 5.1 and 6.0. Further, we corrected for scanner manufacturer to mitigate bias and used only cross-sectional data.

The methods for identifying and calculating regional brain volume and thickness using FreeSurfer have been previously described [41]. Briefly, the volumes of brain regions were corrected for intracranial volume (ICV) by dividing the regions of interest (ROI) of the left and right hemispheres by the ICV. In the following, ICV-corrected bilateral ROI volumes were averaged; a similar approach was adopted for ROI thicknesses. Only MRI scans that exhibited exceptional overall segmentation and successfully passed ADNI’s quality assessment were included. We only included FreeSurfer information with no missing values for each participant. In total, volume information on 46 bilateral brain regions and average thickness information on 34 bilateral brain regions (see Table S3) served as input for our XGBoost classification model.

### CSF biomarker

AD’s neuropathology involves the accumulation of Aβ, leading to plaques, and pTau, resulting in neurofibrillary tangles [45, 46]. We included CSF samples obtained through a fully automated Elecsys® protocol for Aβ and pTau measurements related to MRI acquisition dates to comprehend their association with structural degeneration and diagnostic classification. Aβ 1–42 and pTau181 values were extracted for each participant. It is important to note that the Elecsys® protocols are still being developed, and the reported results are restricted to a specific technical limit (> 1700 pg/mL). Higher values beyond this limit were extrapolated from the calibration curve solely for research purposes, not diagnostics (for additional information on CSF draws and analyses see http://adni.loni.usc.edu).

In this study, we included both proteins in the classification models as a previously established pTau/Aβ ratio to depict the pathological processes of both proteins, demonstrating high concordance with PET measures and clinical diagnoses [47]. Analysis 1 involved CSF, analysis 2 was run without CSF due to the limited number of participants with CSF data (*n* = 32, Table S1), and analysis 3 was run with and without CSF since CSF data were available only for a limited number of participants (*n* = 88, Table S1).

### APOE genotyping

The Apolipoprotein E (APOE) gene’s ε4 allele is widely recognized as the most influential genetic risk factor for non-familial AD [48], and it is related to the grey matter atrophy in AD spectrum [49]. To better understand the diagnostic classification relying on structural ROIs, we incorporated APOE4 genotyping as a variable, factoring in the count of APOE4 alleles (0, 1 or 2) [50]. A blood sample was collected during the screening or baseline visit for APOE genotyping, and the analysis of genotypes followed standard procedures [51].

### Neuropsychological assessment

The participants’ cognitive performance was investigated using a comprehensive neuropsychological test battery. Here, we included the validated memory (MEM) and executive function assessments, as determined by confirmatory factor analysis [52, 53]. The memory scores encompassed the Alzheimer’s Disease Assessment Scale, Logical Memory test, MMSE, and Rey Auditory Verbal Learning Test (RAVLT). The executive function scores were derived from the Category Fluency, Digit Span Backwards, Digit Symbol Substitution, Trails A and B, and Clock Drawing tests [52, 53]. Furthermore, to gain a more comprehensive understanding of the participants’ cognitive profiles, we also included the Montreal-Cognitive-Assessment (MoCA), the Sum of Boxes in the Clinical Dementia Rating Scale (CDRSB), and the Alzheimer’s Disease Assessment Scale Cognitive (ADAS-Cog 13) (see specific details below).

### Ensemble model: XGBoost

For binary and multi-class classification, XGBoost version 1.6.2 (https://xgboost.readthedocs.io/en/latest/python/) was used [54] in Python version 3.9.12 [55] and scikit-learn version 1.0.2 [56] on Jupyter Notebook version 6.4.12 [57] running on Windows 11. XGBoost is a gradient-boosting-based algorithm that uses decision trees as its base model [54], and it has been recently shown to perform well in various regression and classification tasks, including predicting brain age and classifying brain tumors [58–60]. Splitting data into training and testing sets is a common practice in machine learning to assess a trained model’s performance on unseen test datasets. Here, the training and testing data sets were defined separately for each analysis. Specifically, XGBoost models were trained on 65% (training data set) of the stratified data and then evaluated on the remaining 35% (testing data set). A grid search was performed to optimize the hyper parameters of an XGBoost model by using a fivefold cross-validation, which included imaging data (volume and thickness), CSF, demographics (age and sex), and genotyping features as inputs. Optimizing hyperparameters aims to find the best set of hyper parameters resulting in high performing XGBoost models. Balanced accuracy was used as the scoring metric for multi-class classification analysis that comprises imbalanced data. Balanced accuracy accounts for imbalanced class distributions, i.e., it gives equal weight to each class regardless of the number of samples within each class [61, 62]. Furthermore, the scoring metric accuracy was used for binary classification analyses that comprise the balanced data. Finally, SHAP version 0.41.0 (https://shap.readthedocs.io/en/latest/) was used to calculate and visualize the contribution of each input feature to the XGBoost binary and multi-class model’s prediction [63].

In machine learning, precision, recall, and F1-score are metrics used to evaluate the performance of classification models. The F1 score, a metric combining precision and recall, is vital for evaluating classification models as it balances their ability to classify positive instances and minimize false positives correctly. Precision measures the accuracy of positive predictions, while recall evaluates the model’s capability to capture all positive instances, collectively providing a comprehensive evaluation of model performance. Additionally, global accuracy, which assesses the overall correctness of classifications across all classes, offers an overall view of model effectiveness. The error rates were calculated as 1 – global accuracy [64–66]. Post hoc analyses were carried out on the whole sample of each analysis (including training and testing data).

### ADNI dataset classification

We conducted three analyses based on the subjects’ diagnostic status provided by ADNI; individual subjects were only included once in any of the analyses. In the first analysis, a cross-sectional multi-class classification was performed by concatenating subjects with normal cognition (CN), MCI, and AD dementia into one group (*n* = 568). The CSF values of each subject were included as input in the first multi-class classification analysis. In the second analysis, a longitudinal binary classification was performed by concatenating CN stable and CN-to-MCI converters into one group (*n* = 92); here, no CSF values were included as input due to the low number of available data (see above). Third, a longitudinal binary classification was carried out by concatenating the MCI stable and MCI-to-AD converters into one group; here, we performed one analysis with (*n* = 88) and one without CSF (*n* = 378). As mentioned above, all three analyses included brain volume and thickness, demographics, and genetics (APOE4).

The optimized hyperparameters for the first analysis were gamma = 0.01, learning_rate = 0.05, max_depth = 3, min_child_weight = 3, n_estimators = 250, colsample_bytree = 1, and subsample = 0.7; for the second analysis: gamma = 0, learning_rate = 0.001, max_depth = 1, min_child_weight = 5, n_estimators = 150, colsample_bytree = 0.7, and subsample = 1; the third analysis with CSF: gamma = 0, learning_rate = 0.01, max_depth = 1, min_child_weight = 3, n_estimators = 200, colsample_bytree = 1, and subsample = 0.5; and for the third without CSF: gamma = 0.3, learning_rate = 0.1, max_depth = 6, min_child_weight = 3, n_estimators = 50, colsample_bytree = 0.7, and subsample = 1.

### Ethics approval and consent to participate

Each center collecting data for ADNI provided an IRB (Institutional Review Board) approval and meets ADNI’s requirements in accordance with the declaration of Helsinki. Informed consent was obtained from all ADNI participants (for more information at http://adni.loni.usc.edu). Further, the analyses presented here were approved by the local Ethics Committee of the University of Lübeck and carried out after ADNI’s recommendations including the approval of the manuscript before submitting to a journal.

## Results

### Diagnoses and neuropsychological tests

For each of the three analyses, one-way ANCOVAs on the neuropsychological assessments, corrected for age, sex, and education (in years), were carried out. As expected, in cross-sectional analysis 1, significant differences emerged between CN, MCI, and AD dementia (all tests *p* < 0.05/n, *n* = 7 tests) with significantly lower cognitive performance in MCI and AD dementia (Table S2). In longitudinal analysis 2, the CN-to-MCI converters converted after, on average, 15 months (SD = 8.35) and the CN stables remained stable for, on average, 70.54 months (SD = 47.94). The CN-to-MCI converters scored lower on ADNI MEM (*F*(1,87) = 9.88, *p* = 0.002, Table S2) and ADAS-Cog 13 (*F*(1,87) = 14.44, *p* < 0.001, Table S2); however, for the other cognitive assessments, there were no significant differences (*p* < 0.05/n, *n* = 7 tests, Table S2). In longitudinal analysis 3, MCI-to-AD converters converted, on average, after 9.53 months (SD = 5.85), and the MCI stables remained stable for, on average, 52.5 months (SD = 93.5). The MCI-to-AD converters had lower scores in all neuropsychological assessments compared to MCI stable (Table S2).

### ADNI dataset classification results

**Fig. 2:**
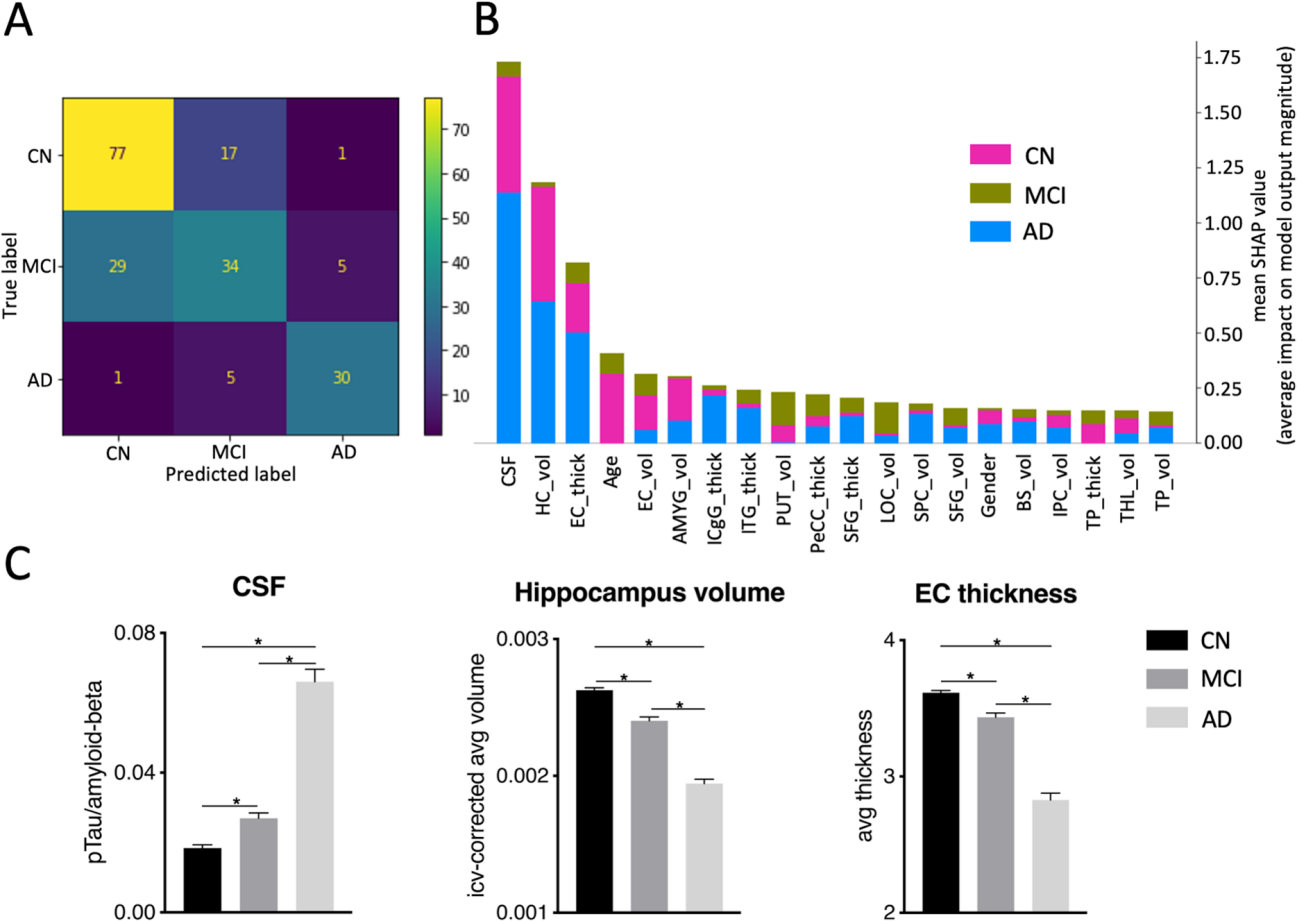
Analysis 1, cross-sectional multiclass classification of CN vs MCI vs AD dementia. **A** Displays the confusion matrix of the testing data set (*n* = 199), visually representing the accuracy and misclassification of predictions among the groups. **B** SHAP plot highlighting key features and their respective impact magnitude on class predictions based on the testing data set. **C** Displays the three dominant features for the entire sample (*n* = 568): CSF status, hippocampus volume and entorhinal cortex thickness. Hippocampus volume and entorhinal thickness was significantly lower, and CSF ratio was significantly higher, across the three groups. *Significant after Bonferroni correction *p* < *0.05/n* (*n* = 9 tests). Abbreviations: SHAP, SHapley Additive exPlanations; CSF, cerebrospinal fluid; HC_vol, hippocampus volume; EC_thick, enthorinal cortex thickness; AMYG_vol, amygdala volume; ICgG_thick, isthmus of cingulate gyrus thickness; ITG_thick, inferior temporal gyrus thickness; PUT_vol, putamen volume; PeCC_thick, pericalcarine cortex thickness; SFG_thick, superior frontal gyrus thickness; LOC_vol, lateral occipital cortex volume; SPC_vol, superior parietal cortex volume; SFG_vol, superior frontal gyrus volume; BS_vol, brain stem volume; IPC_vol, inferior parietal cortex volume; TP_thick, temporal pole thickness; THL_vol, thalamus volume; TP_vol, temporal pole volume

XGBoost model performances for each analysis are reported in Table 1. For analysis 1 (CN vs MCI vs AD dementia, Fig. 2A), the XGBoost model for the cross-sectional multi-class classification achieved an overall global accuracy (i.e., classification accuracy) of 71%, and the model’s f1-scores were 76% for CN, 55% for MCI, and 83% for AD dementia. SHAP revealed the following features with the highest predictive value in the classification: CSF status, hippocampus volume, and entorhinal cortex thickness (Fig. 2B). While the hippocampus volume and entorhinal cortex thickness were significantly lower, the CSF status was significantly higher (*p* < 0.05/n, *n* = 9 tests) across the three groups in the entire dataset, as confirmed by post hoc *t*-tests or Mann–Whitney *U* tests (when normal distribution was not given; Fig. 2C, Table S4).

**Table 1:** XGBoost model performance for each analysis

	precision	recall	f1-score	Macro average	global accuracy of the model	Error Rate	N_Test_
*Analysis 1 (cross-sectional, CN vs MCI vs AD, with CSF status)*							
CN	72%	81%	76%	71%	71%	29%	95
MCI	61%	50%	55%				68
AD	83%	83%	83%				36
*Analysis 2* (CN stable vs. CN-to-MCI converters, *without CSF status*)							
CN stable	72%	81%	76%	76%	76%	24%	16
CN-to-MCI converters	80%	71%	75%				17
*Analysis 3* (MCI stable vs MCI-to-AD converters, *with CSF status*)							
MCI stable	74%	88%	80%	77%	77%	23%	16
MCI-to-AD converters	83%	67%	74%				15
*Analysis 3* (MCI stable vs MCI-to-AD converters, *without CSF status*)							
MCI stable	68%	74%	71%	70%	70%	30%	66
MCI-to-AD converters	72%	66%	69%				67

The precision, recall, f1-score, macro-average, global accuracy of the model, and N_Test_ (the number of subjects included in the testing set) or each analysis separately. Abbreviations: *CN*, cognitive normal; *MCI*, mild cognitive impairment; *AD*, Alzheimer’s disease dementia; *CSF*, cerebro spinal fluid

**Fig. 3:**
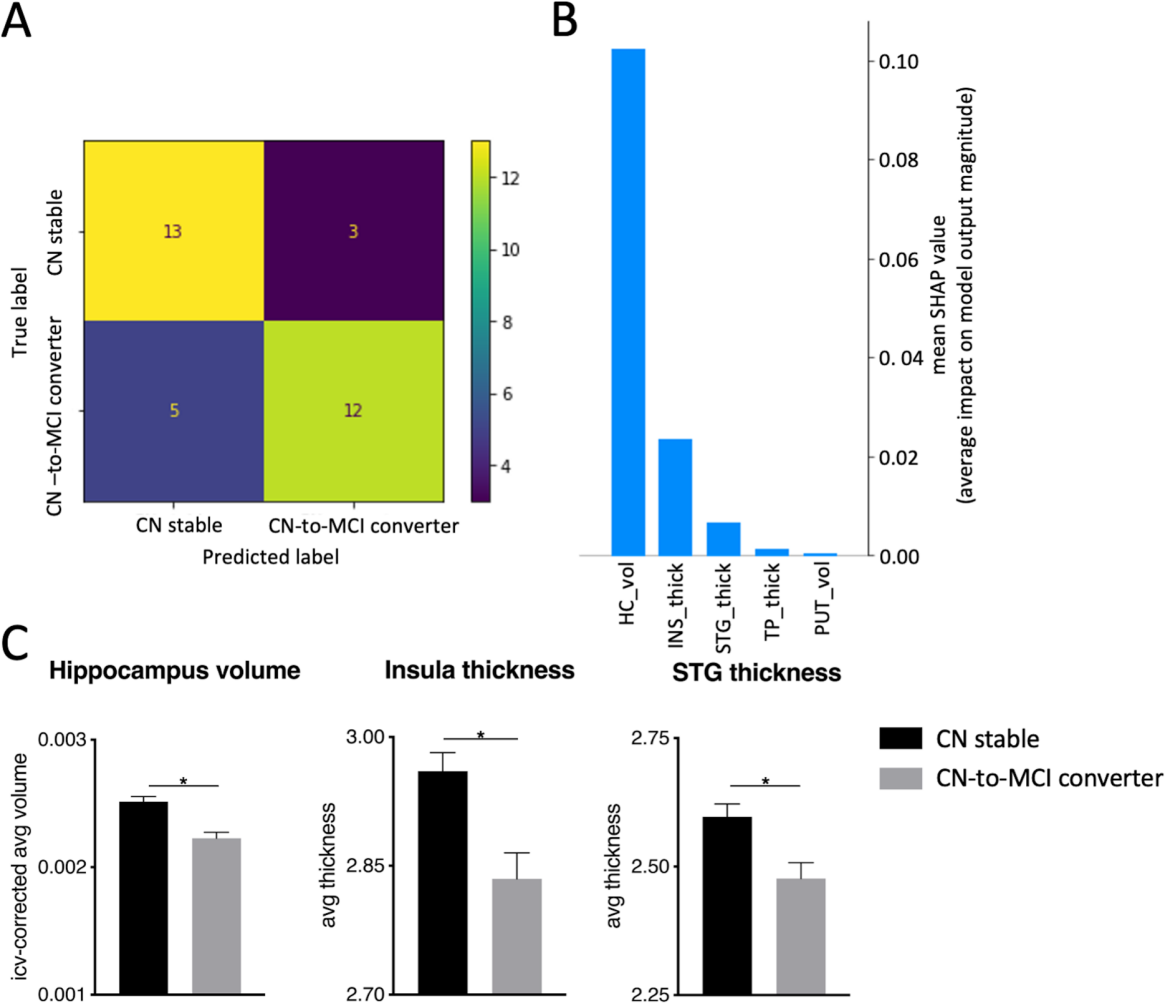
Analysis 2, longitudinal binary-class classification on the testing data of CN stable vs CN-to-MCI converters. **A** Displays the confusion matrix, visually representing the accuracy and misclassification of predictions among the groups for the testing data set (*n* = 33). **B** SHAP plot highlighting key features and their respective impact magnitude on class predictions based on the testing data set. **C** Showing bar plots of the three dominant features for the entire sample (*n* = 92): hippocampus volume; insula thickness; superior temporal gyrus (STG) thickness. All regions showed significantly lower values in the CN-to-MCI converter group. *Significant after Bonferroni correction *p* < 0.05/*n* (*n* = 3 tests). Abbreviations: SHAP, SHapley Additive exPlanations; HC_vol, hippocampus volume; INS_thick, insula thickness; TP_vol, temporal pole volume; PUT_vol, putamen volume

For analysis 2 (CN stable vs. CN-to-MCI converters, Fig. 3A), the XGBoost model for longitudinal binary-class classification achieved an overall global accuracy of 76%, and the model’s f1-scores were 76% for CN stable and 75% for CN-to-MCI converters. SHAP revealed the following features with the highest predictive value in the classification task: hippocampus volume, insula thickness, and superior temporal gyrus thickness (Fig. 3B). These brain regions showed lower values (i.e., volume and thickness, respectively) in CN-to-MCI converters compared to CN stable (Fig. 3C) as confirmed by post hoc *t*-tests (*p* < 0.05/n, *n* = 3 tests) calculated on the entire sample (Table S4).

**Fig. 4:**
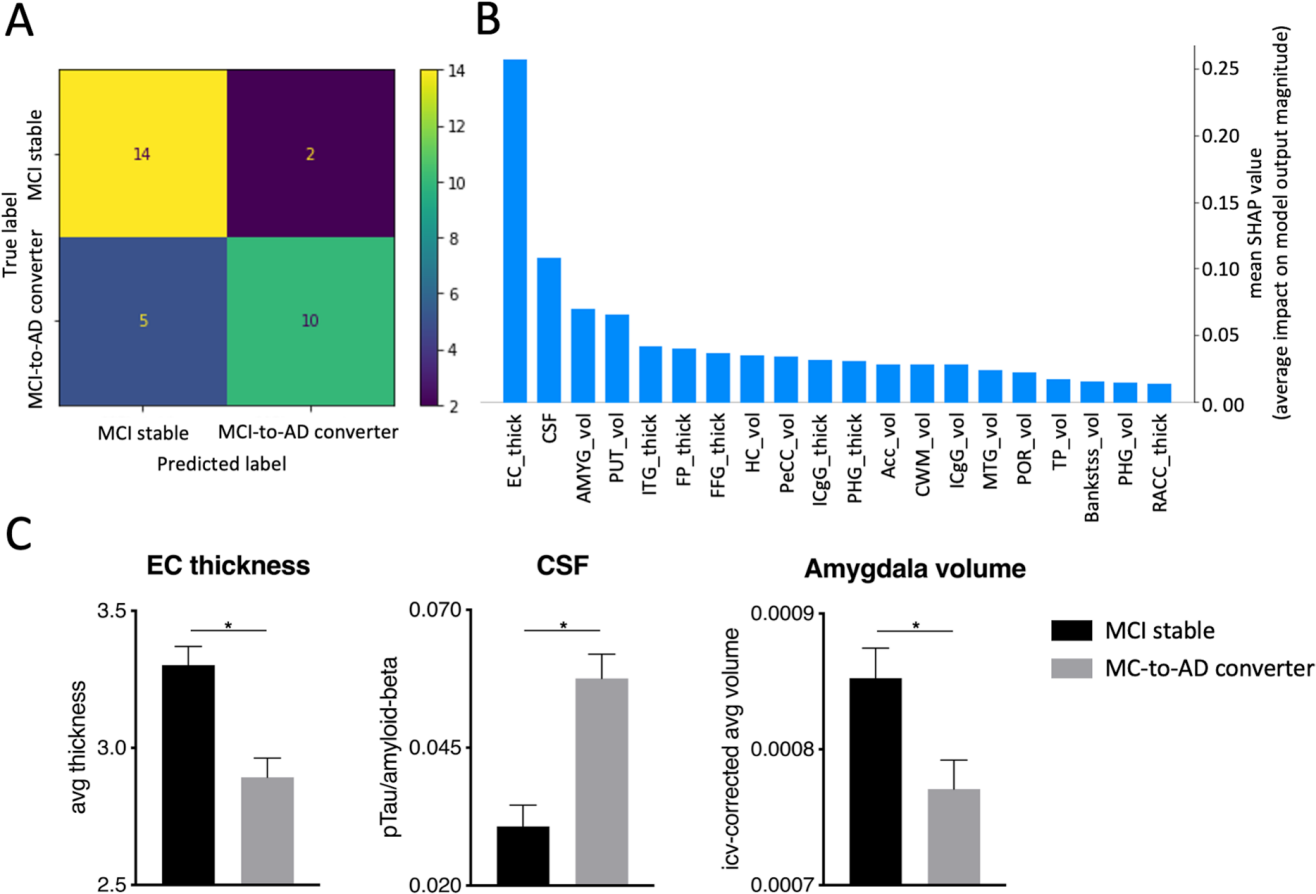
Analysis 3, longitudinal binary-class classification on the testing data of MCI stable vs MCI-to-AD converters, including CSF status. **A** displays the confusion matrix of the testing data set (*n* = 31), visually representing the accuracy and misclassification of predictions among the groups. **B** features a SHAP plot highlighting key features and their respective impact magnitude on class predictions based on the testing data set. **C** shows the three dominant features in bar plots for the entire sample (*n* = 88): entorhinal cortex thickness, CSF status, and amygdala volume. Entorhinal cortex thickness and amygdala volume were significantly lower in MCI-to-AD converters, and the CSF ratio was significantly higher. *Significant after Bonferroni correction *p* < 0.05/*n* (*n* = 3 tests). Abbreviations: SHAP, SHapley Additive exPlanations; CSF, Cerebrospinal Fluid; EC_thick, enthorinal cortex thickness; AMYG_vol, amygdala volume; PUT_vol, putamen volume; ITG_thick, inferior temporal gyrus thickness; FP_thick, frontal pole thickness; FFG_thick, fusiform gyrus thickness; HC_vol, hippocampus volume; PeCC_vol, pericalcarine cortex volume; ICgG_thick, isthmus of cingulate gyrus thickness; PHG_thick, parahippocampal gyrus thickness; ACC_vol, accumbens area volume; CWM_vol, cerebellum white matter volume; ICgG_vol, isthmus of cingulate gyrus volume; MTG_vol, middle temporal gyrus volume; POR_vol, pars orbitalis volume; TP_vol, temporal pole volume; Bankstss_vol, banks of the superior temporal sulcus volume; PHG_vol, parahippocampal gyrus volume; RACC_thick, rostral anterior cingulate cortex thickness

**Fig. 5:**
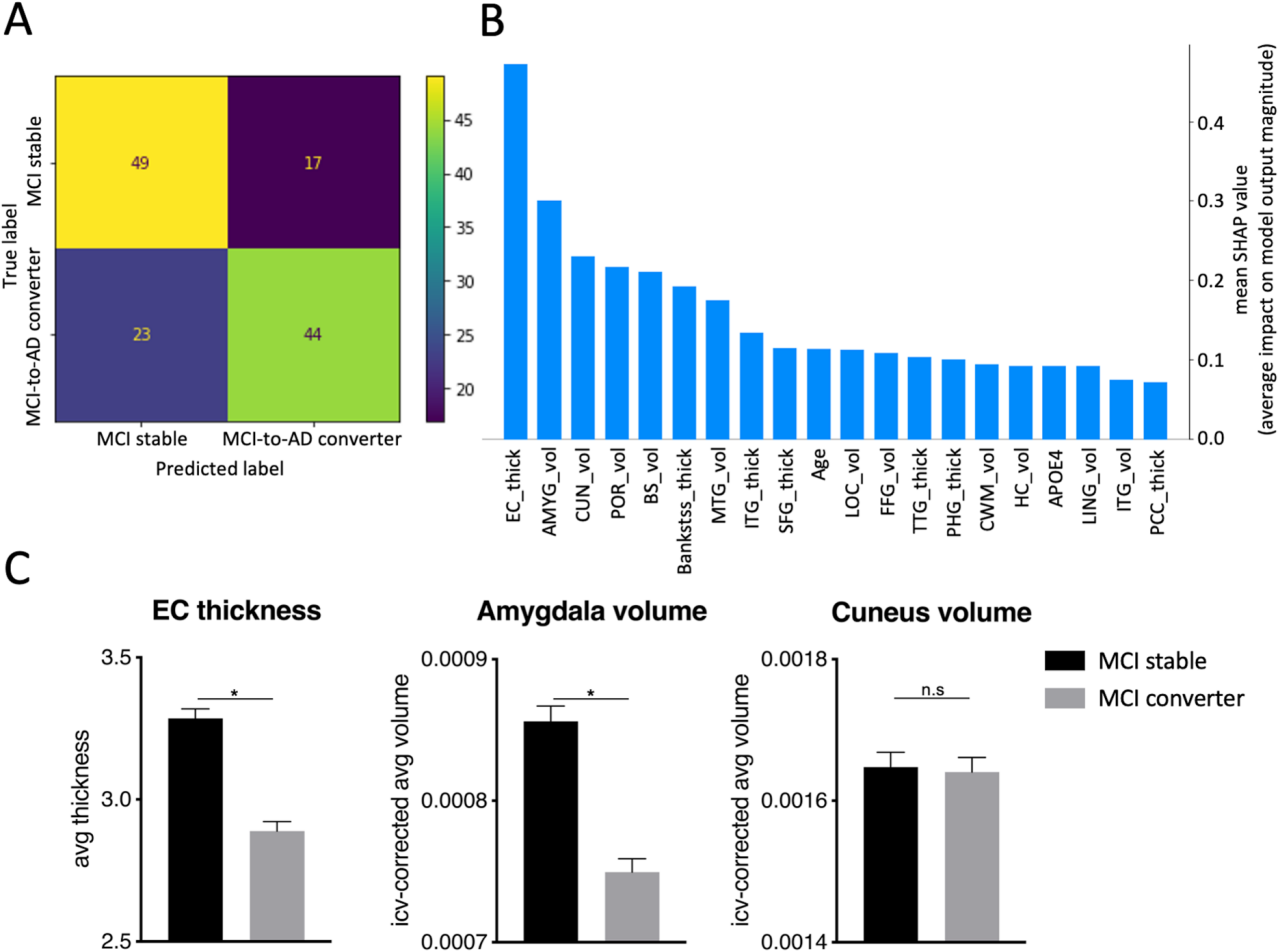
Analysis 3, longitudinal classification of MCI stable vs MCI-to-AD converters, *not* including CSF. **A** displays the confusion matrix on the testing data (*n* = 133), visually representing the accuracy and misclassification of predictions among the groups. **B** SHAP plot highlighting key features and their respective impact magnitude on class predictions based on the testing data set. **C** shows the bar plots of the three dominant features: entorhinal cortex thickness, amygdala volume, and cuneus volume. Apart from the cuneus, all regions show significant reductions in MCI-to-AD converters. *Significant after Bonferroni correction *p* < 0.05/*n* (*n* = 3 tests). Abbreviations: SHAP, SHapley Additive exPlanations; EC_thick, enthorinal cortex thickness; AMYG_vol, amygdala volume; CUN_vol, cuneus volume; POR_vol, Pars orbitalis_volume; BS_vol, brainstem volume; Bankstss_thick, banks of the superior temporal sulcus thickness; MTG_vol, middle temporal gyrus volume; ITG_thick, inferior temporal gyrus thickness; SFG_thick, superior frontal gyrus thickness; LOC_vol, lateral occipital cortex volume; FFG_vol, fusiform gyrus volume; TTG_thick, transverse temporal gyrus thickness; PHG_thick, parahippocampal gyrus; CWM_vol, cerebellum white matter volume; HC_vol, hippocampus volume; APOE4, apolipoprotein E4; LING_vol, lingual gyrus volume; ITG_vol, inferior temporal gyrus volume; PCC_thick, posterior cingulate cortex thickness

For analysis 3 (MCI stable vs MCI-to-AD converters, Fig. 4A), the XGBoost model for longitudinal binary-class classification achieved an overall global accuracy of 77%, and the model’s f1-score were 80% for the MCI stable and 74% for the MCI-to-AD converters. SHAP revealed the following features with the highest predictive value in the classification task: entorhinal cortex thickness, CSF status, and amygdala volume (Fig. 4B). Again, the brain features showed lower and the CSF status higher values in MCI-to-AD converters compared to MCI stable, further supported by post hoc *t*-tests or Mann–Whitney *U* tests (*p* < 0.05/n, *n* = 3 tests) calculated on the entire sample (Fig. 4C, Table S4). Finally, the same XGBoost model for binary-class classification without CSF status (Fig. 5A) showed an overall global accuracy of 70%, and the model’s f1-score was 71% for MCI stable and 69% for MCI-to-AD converters. SHAP revealed the following features with the highest predictive value in the classification task: entorhinal cortex thickness, amygdala volume, and cuneus volume (Fig. 5B). Again, these features showed lower values in MCI-to-AD converters compared to MCI stable, confirmed by post hoc *t*-tests or Mann–Whitney *U* tests on the entire sample (*p* < 0.05/n, *n* = 3 tests) (Fig. 5C, Table S4) (see Fig. 1D for a depiction of the most relevant brain regions across our analyses).

## Discussion

We investigated the underlying principles of AD progression on the basis of longitudinal diagnostic information, structural MRI data, APOE4, CSF status, and sociodemographics from healthy older adults, individuals with MCI and patients with AD dementia. Using a state-of-the-gradient-boosting based machine learning algorithm, we can show that a multi-class classification of CN vs. MCI vs. AD dementia is prominently driven by CSF status (i.e., proteinopathy), hippocampal volume, and entorhinal thickness, which confirms previous work and demonstrates the feasibility of our approach. As a novel main observation, the accurate classification of a conversion from CN to MCI was mainly driven by hippocampal volume followed by thickness of the insula and superior temporal gyrus; the accurate classification of a conversion from MCI to AD dementia was primarily based on thickness of the entorhinal cortex followed by CSF status and amygdala volume. While this indicates a more prominent role of the hippocampus in the conversion from CN to MCI and entorhinal cortex from MCI to AD dementia, our findings also demonstrate that, in comparison, other brain regions are less important for the classification. Therefore, our findings give novel insights into the developmental trajectories of AD, and they highlight novel ways to employ machine learning in the context of neuropsychiatric disorders.

Our cross-sectional multi-class classification of CN, MCI, and AD dementia achieved a global accuracy of 71%, which is comparable with previous research showing similar accuracies ranging from 59 to 77% [15–17]. Importantly, the three most relevant features of the classification were CSF ratio, hippocampal volume, and entorhinal thickness, followed by age and other features with comparably much lower predictive value (Fig. 2B). With regard to proteinopathy, Aβ and pTau have long been associated with AD [67], and individual CSF status can be superior compared to other features derived from structural MRI, FDG-PET, and APOE [50, 68]. This might be due to the fact that CSF biomarkers represent distinct biochemical aspects of AD pathology [69] and provide complementary information to structural MRI measures [50]. Therefore, abnormalities in CSF biomarkers, specifically Aβ, can precede neurodegeneration, including structural MRI changes and cognitive impairment [5, 7], which is compatible with our observation of a reliable cross-sectional classification driven by CSF status [70]. Importantly, our classification approach (in all analyses) was based on a combination of several features, which appears to be the most reliable method of classifying the AD spectrum [50]. Along these lines, hippocampal volume and entorhinal cortex thickness were the second and third most prominent features that contributed to a reliable cross-sectional classification. Specifically, both brain regions showed more pronounced volume and thickness, respectively, in CN, followed by MCI and AD dementia, which resembles the disease progression (Fig. 2C, Table S4) [71, 72]. It also aligns with past studies using machine learning, in which hippocampal features and the entorhinal cortex strongly contribute to AD classification [17]. Finally, previous VBM studies comparing CN vs. AD dementia also revealed significant differences in grey matter within the MTL, including hippocampus [73–75], entorhinal cortex [76–78], and amygdala [79, 80], which underlines the importance of medial temporal lobe brain regions in the development of AD, which will be discussed in more detail below.

Note that precision and recall in the cross-sectional multiclass classification of all three groups, MCI was associated with the lowest model’s accuracy (f1-score of 55%, Table 1), followed by cognitively normal older adults (76%, Table 1) and then AD dementia patients (83%, Table 1). This finding is compatible with previous reports of lower classifier performance for MCI [13, 15] and can be explained by the notion that MCI is clinically heterogeneous with distinct but less pronounced brain atrophy compared to AD dementia [13]. In a similar vein, it is important to note that our machine learning approach does not compare the effects of different features but combines them resulting in degrees of classifications.

Cross-sectional classifications can only portray group differences, but they cannot characterize longitudinal or developmental changes. Together with the fact that not all healthy older adults develop MCI, and not all MCI patients progress to AD dementia, it is important to understand the underlying and contributing factors [81]. Here, our longitudinal binary classification of CN-to-MCI converters vs. CN stable revealed the hippocampus volume in particular, but also the thickness of the insula and superior temporal gyrus (Fig. 3B), as the three most prominent features contributing to an accurate classification. In all three regions, healthy older adults that subsequently developed MCI showed a significantly lower volume and thickness, respectively, compared to those who remained CN and therefore did not develop MCI (Fig. 3C, Table S4). While this highlights the importance of the medial temporal lobe in the development of MCI, it is interesting to note that only the hippocampus but not the entorhinal cortex emerged as significant feature in our analysis [82, 83]. While this could be due to the rather low number of subjects in this analysis (*n* = 46 in each group), our classification model reflected a global accuracy of 76%, which is comparable with previous work reporting similar accuracies between 70 and 78% [43, 82, 83].

The contribution of the insula and superior temporal gyrus, with lower thicknesses in CN-to-MCI converters, is in line with cross-sectional VBM study results showing lower gray matter thickness in CN compared to MCI [84]. While cortical thinning originates in temporal brain regions [84–86] (see below) and is associated with neuropsychological performance [84], the role of the insula is less clear. However, as AD advances, the gyrification in the insular cortex decreases from MCI to more severe AD dementia, while folding complexity was linked to better memory performance specifically in AD dementia [87]. Furthermore, with AD progression the insula is affected by proteinopathies [88] and structural degeneration, which impacts various functions such as olfaction [89], gustation, anatomic self-control, self-awareness, and emotion, possibly contributing to the diverse symptoms of AD [88, 90, 91].

Compatible with the observation of reduced hippocampal volumes, CN-to-MCI converters showed significantly worse memory functioning (MEM). Indeed, the hippocampus plays a central role in learning and memory with declines in structure and function even in healthy older adults [92]. While there were no significant differences in executive functioning or the MMSE, performances in other tests, however, point towards rather global differences in cognitive abilities (Table S2). For instance, the MoCA, which was lower in CN-to-MCI converters, quantifies several cognitive domains, including visuospatial/executive abilities, language and verbal abilities, memory as well as attention [93]. Importantly, both groups showed, on average, MoCA values higher than a cutoff value of 22 indicating their normal cognitive abilities [93]. Therefore, both groups are, from a clinical perspective, cognitively unimpaired, and they do not show significant brain atrophy; yet, healthy older adults who later develop MCI show lower grey matter volume, especially in the hippocampus, together with lower scores in several cognitive tests. While this observation does not allow a clear conclusion on whether structural degeneration precedes cognitive decline, which has been suggested before [5, 7], they clearly show that both factors—hippocampal volume and cognitive abilities—can provide significant biomarkers to predict developmental trajectories in cognitively normal older adults. Therefore, this finding is of high clinical relevance and adds novel insights to the limited number of studies using machine learning on the conversion from CN to MCI [43, 82, 83, 94, 95].

Our longitudinal binary classification with MCI patients who converted (MCI-to-AD converters) and MCI patients who remained stable (MCI stable) had global accuracies ranging from 70% (excluding CSF, Table 1) to 77% (with CSF, Table 1), which is comparable to others reporting accuracies ranging from 74 to 89% [96]. In our case, entorhinal cortex thickness and amygdala volume were the two most prominent features in both classification scenarios, with more pronounced reductions in MCI-to-AD converters compared to MCI stable (Fig. 4B and C and Fig. 5B and C, Table S4). The entorhinal cortex is considered a critical relay for the communication between the neocortex and hippocampus, and, therefore, plays an important role in several cognitive processes, including learning and memory [97, 98]. With regard to AD progression, the entorhinal cortex appears to be one of the first brain regions affected, which could lead to memory impairments. For instance, deposits of Aβ and p-tau first occur in the trans-entorhinal and entorhinal cortex, which is followed by the hippocampus and other cortical regions [99]. MRI studies confirm this picture by demonstrating that volume [100] and shape [101] of the hippocampus as well as hippocampal subfields [102] differ as a function of disease progression (for a review see [103]). There are similar but more heterogeneous effects in the entorhinal cortex and the amygdala [104], and therefore, it is not surprising that both brain regions were the best predictors for the conversion from MCI to AD dementia in our study.

Interestingly, when analysis 3 was run solely on participants where CSF drawings were available, the entorhinal cortex still emerged as the most predictive feature followed by CSF status (Fig. 4B), which could have important implications for the clinical context. In fact, whole-brain MRIs are much less invasive than CSF drawings and bear a much lower risk of potential side effects. Physiologically, this possibly reflects a stronger atrophy of the entorhinal cortex as compared to CSF-based accumulations of proteinopathies in the disease progression. In other words, while Aβ and p-tau accumulations are particularly pronounced in the early stages, this could be followed by more pronounced neurodegeneration of the entorhinal cortex in the following stages from MCI to AD dementia, which is compatible with the AT(N) framework [7]. The AT(N) framework is based on Aβ (A), tau (T), and neurodegeneration (N) to categorize distinct disease stages of AD and to investigate the interaction of the pathological processes. However, cutoff values—often determined by PET and in some studies CSF—are not well defined [7] and large sample sizes are required to account for at least three groups (A-T-, A + T-, A + T +) [105]. While in our study the AT(N) framework could help to exclude other pathological conditions affecting the MTL (e.g., limbic-predominant age-related TDP- 43 encephalopathy (LATE), primary age-related tauopathy (PART), Lewy body dementia (LBD), frontotemporal dementia (FTD)), our work aligns more closely with clinical practice and ADNI by using neuropsychological assessments to diagnose MCI and AD dementia (see Methods).

The model without CSF status revealed the cuneus (Fig. 5B) as third most relevant contributing feature, which is in line with previous classifications of MCI-to-AD converters vs. MCI stable [106, 107]. Indeed, the cuneus is associated with processing visual information, integrating sensory information, and cognitive processes including attention, learning, and memory [108, 109], and its atrophy has been associated with an increased risk of AD [102]. However, in a post-hoc analysis, the cuneus did not show significant volume differences in MCI-to-AD converters compared to MCI-stable individuals. This might be explained by differences in the underlying statistical principles of both analyses and indicates the need for a careful interpretation.

Finally, we would like to point out the following strengths and limitations. First, our study included a rather large sample of well characterized subjects from the ADNI database. Some analyses, however, were based on smaller numbers (analysis 2 and analysis 3 with CSF assays available), which calls for cautious interpretations. Second, machine learning offers a robust and reliable approach facilitating generalization and optimization [110, 111]. In this regard, XGboost in combination with SHAP allowed us, in an agnostic way, to pinpoint the contributing features [63]. Note, however, that other approaches exist but a direct comparison was beyond the scope of this study (see [112] for more information). Third, our volumetric and thickness data were based on the well-established 2010 Desikan-Killiany atlas [40] and a subcortical segmentation [41, 42]. However, this does not include a precise quantification of some brain regions [41, 42], such as the nucleus basalis of Meynert (NbM), which might play a central role in the progression of AD [9, 10, 81, 113]. Fourth, ADNI data was measured at multiple sites with diverse MRI scanners, which we addressed by only pre-selecting high-quality data and including scanner manufacturers as covariates in our analyses. Fifth, CSF assays offer an important way to investigate the underlying neurobiology of AD, which, in future work, could be complemented by more regionally specific PET data. Sixth, we focused on participants with stable diagnoses and information on the conversion (with rather short conversion times), omitting potential reversions [114]. Seventh, a model with more features, such as clinical, cognitive, genetic, PET, and CSF status, could enhance model accuracies [115], but this would require more data points (i.e., subjects). These aspects, together with alternative but not mutually exclusive frameworks [7], should be considered in future work.

## Conclusion

To conclude, machine learning in combination with structural MRI data, sociodemographic, CSF status, and genetic information allows a precise and explainable classification along the AD continuum. Our findings indicate that the hippocampus plays a prominent role in the conversion from healthy aging to MCI; the entorhinal cortex, on the other hand, contributes more to the conversion from MCI to AD dementia. As such, our study gives novel insights into the developmental trajectories from healthy to pathological aging by suggesting a dissociation of specific medial temporal lobe brain regions. This, in turn, could contribute to earlier and more accurate diagnoses and interventions.

## Supplementary Information

Below is the link to the electronic supplementary material.

Supplementary file1 (DOCX 43.2 KB)

## Data Availability

All data from this study can be obtained freely upon request from the Image and Data Archive (IDA) operated by the Laboratory of Neuro Imaging (LONI) at https://ida.loni.usc.edu. Our analysis code is available at OSF: https://osf.io/ajwct/?view_only=4f2012b70a3b4ea18ac85e847767657d.

## Abbreviations

*Aβ*: Amyloid-β
*APOE*: Apolipoprotein E
*ADAS-Cog*: Alzheimer’s Disease Assessment Scale Cognitive
*ADNI*: Alzheimer’s Disease Neuroimaging Initiative
*CDRSB*: Clinical Dementia Rating Scale
*CN*: Cognitive normal
*ICV*: Intracranial volume
*MCI*: Mild cognitive impairment
*MEM*: Memory
*MMSE*: Mini-Mental-State Examination
*MoCA*: Montreal-Cognitive-Assessment
*NbM*: Nucleus basalis of Meynert
*pTau*: Hyperphosphorylated Tau
*RAVLT*: Rey Auditory Verbal Learning Test
*ROI*: Region of interest
*SHAP*: SHapley Additive exPlanations
*XGBoost*: EXtreme Gradient Boosting

## References

[1] Hedden T, Gabrieli JDE. Insights into the ageing mind: a view from cognitive neuroscience. Nat Rev Neurosci. 2004;5:87–96. 14735112 10.1038/nrn1323

[2] Mesulam M. The cholinergic innervation of the human cerebral cortex. Prog Brain Res. 2004;145:67–78. 14650907 10.1016/S0079-6123(03)45004-8

[3] Biel D, Steiger TK, Bunzeck N. Age-related iron accumulation and demyelination in the basal ganglia are closely related to verbal memory and executive functioning. Sci Rep. 2021;11:9438. 33941809 10.1038/s41598-021-88840-1 PMC8093241

[4] Brossollet I, Gallet Q, Favre P, Houenou J. Machine learning and brain imaging for psychiatric disorders: new perspectives. In: Colliot O, editor. Mach Learn Brain Disord [Internet]. New York, NY: Springer US; 2023 [cited 2023 Sep 8]. p. 1009–36. Available from: 10.1007/978-1-0716-3195-9_32 37988551

[5] Jack CR, Holtzman DM. Biomarker modeling of Alzheimer’s disease. Neuron. 2013;80:1347–58. 24360540 10.1016/j.neuron.2013.12.003 PMC3928967

[6] Olsson B, Lautner R, Andreasson U, Öhrfelt A, Portelius E, Bjerke M, et al. CSF and blood biomarkers for the diagnosis of Alzheimer’s disease: a systematic review and meta-analysis. Lancet Neurol. 2016;15:673–84. 27068280 10.1016/S1474-4422(16)00070-3

[7] Jack CR, Bennett DA, Blennow K, Carrillo MC, Dunn B, Haeberlein SB, et al. NIA-AA Research Framework: Toward a biological definition of Alzheimer’s disease. Alzheimers Dement. 2018;14:535–62. 29653606 10.1016/j.jalz.2018.02.018 PMC5958625

[8] Beardmore R, Hou R, Darekar A, Holmes C, Boche D. The locus coeruleus in aging and Alzheimer’s disease: a postmortem and brain imaging review. Ferreira S, editor J Alzheimers Dis. 2021;83:5–22. 10.3233/JAD-210191 PMC8461706 34219717

[9] Fernández-Cabello S, Kronbichler M, Van Dijk KRA, Goodman JA, Spreng RN, Schmitz TW, et al. Basal forebrain volume reliably predicts the cortical spread of Alzheimer’s degeneration. Brain. 2020;143:993–1009. 32203580 10.1093/brain/awaa012 PMC7092749

[10] Schmitz TW, Spreng RN. Basal forebrain degeneration precedes and predicts the cortical spread of Alzheimer’s pathology. Nat Commun. 2016;7:13249. 27811848 10.1038/ncomms13249 PMC5097157

[11] Counts SE, Ikonomovic MD, Mercado N, Vega IE, Mufson EJ. Biomarkers for the early detection and progression of Alzheimer’s disease. Neurotherapeutics. 2017;14:35–53. 27738903 10.1007/s13311-016-0481-z PMC5233625

[12] Rathore S, Habes M, Iftikhar MA, Shacklett A, Davatzikos C. A review on neuroimaging-based classification studies and associated feature extraction methods for Alzheimer’s disease and its prodromal stages. Neuroimage. 2017;155:530–48. 28414186 10.1016/j.neuroimage.2017.03.057 PMC5511557

[13] Basaia S, Agosta F, Wagner L, Canu E, Magnani G, Santangelo R, et al. Automated classification of Alzheimer’s disease and mild cognitive impairment using a single MRI and deep neural networks. NeuroImage Clin. 2019;21:101645. 30584016 10.1016/j.nicl.2018.101645 PMC6413333

[14] Sarica A, Cerasa A, Quattrone A. Random forest algorithm for the classification of neuroimaging data in Alzheimer’s disease: a systematic review. Front Aging Neurosci. 2017;9:329. 29056906 10.3389/fnagi.2017.00329 PMC5635046

[15] Pellegrini E, Ballerini L, Hernandez MDCV, Chappell FM, González-Castro V, Anblagan D, et al. Machine learning of neuroimaging for assisted diagnosis of cognitive impairment and dementia: a systematic review. Alzheimers Dement Diagn Assess Dis Monit. 2018;10:519–35. 10.1016/j.dadm.2018.07.004 PMC6197752 30364671

[16] Tanveer M, Richhariya B, Khan RU, Rashid AH, Khanna P, Prasad M, et al. Machine learning techniques for the diagnosis of Alzheimer’s disease: a review. ACM Trans Multimed Comput Commun Appl. 2020;16:1–35.

[17] Diogo VS, Ferreira HA, Prata D, for the Alzheimer’s Disease Neuroimaging Initiative. Early diagnosis of Alzheimer’s disease using machine learning: a multi-diagnostic, generalizable approach. Alzheimers Res Ther. 2022;14:107. 35922851 10.1186/s13195-022-01047-y PMC9347083

[18] Böhle M, Eitel F, Weygandt M, Ritter K. Layer-wise relevance propagation for explaining deep neural network decisions in MRI-based Alzheimer’s disease classification. Front Aging Neurosci. 2019;11:194. 31417397 10.3389/fnagi.2019.00194 PMC6685087

[19] Son S-J, Kim J, Park H. Structural and functional connectional fingerprints in mild cognitive impairment and Alzheimer’s disease patients. Fan Y, editor. PLOS ONE. 2017;12:e0173426. 28333946 10.1371/journal.pone.0173426 PMC5363868

[20] Rondina JM, Ferreira LK, De Souza Duran FL, Kubo R, Ono CR, Leite CC, et al. Selecting the most relevant brain regions to discriminate Alzheimer’s disease patients from healthy controls using multiple kernel learning: a comparison across functional and structural imaging modalities and atlases. NeuroImage Clin. 2018;17:628–41. 29234599 10.1016/j.nicl.2017.10.026 PMC5716956

[21] Pan D, Zeng A, Jia L, Huang Y, Frizzell T, Song X. Early detection of Alzheimer’s disease using magnetic resonance imaging: a novel approach combining convolutional neural networks and ensemble learning. Front Neurosci. 2020;14:259. 32477040 10.3389/fnins.2020.00259 PMC7238823

[22] Grueso S, Viejo-Sobera R. Machine learning methods for predicting progression from mild cognitive impairment to Alzheimer’s disease dementia: a systematic review. Alzheimers Res Ther. 2021;13:162. 34583745 10.1186/s13195-021-00900-w PMC8480074

[23] Devanand DP, Pradhaban G, Liu X, Khandji A, De Santi S, Segal S, et al. Hippocampal and entorhinal atrophy in mild cognitive impairment: prediction of Alzheimer disease. Neurology. 2007;68:828–36. 17353470 10.1212/01.wnl.0000256697.20968.d7

[24] Westman E, Muehlboeck J-S, Simmons A. Combining MRI and CSF measures for classification of Alzheimer’s disease and prediction of mild cognitive impairment conversion. Neuroimage. 2012;62:229–38. 22580170 10.1016/j.neuroimage.2012.04.056

[25] Killiany RJ, Hyman BT, Gomez-Isla T, Moss MB, Kikinis R, Jolesz F, et al. MRI measures of entorhinal cortex vs hippocampus in preclinical AD. Neurology. 2002;58:1188–96. 11971085 10.1212/wnl.58.8.1188

[26] Yeung L-K, Hale C, Rizvi B, Igwe K, Sloan RP, Honig LS, et al. Anterolateral entorhinal cortex volume is associated with memory retention in clinically unimpaired older adults. Neurobiol Aging. 2021;98:134–45. 33278686 10.1016/j.neurobiolaging.2020.10.031 PMC7870549

[27] Knopman DS, Lundt ES, Therneau TM, Vemuri P, Lowe VJ, Kantarci K, et al. Entorhinal cortex tau, amyloid-β, cortical thickness and memory performance in non-demented subjects. Brain J Neurol. 2019;142:1148–60. 10.1093/brain/awz025 PMC6439321 30759182

[28] Konishi K, Joober R, Poirier J, MacDonald K, Chakravarty M, Patel R, et al. Healthy versus entorhinal cortical atrophy identification in asymptomatic APOE4 carriers at risk for Alzheimer’s disease. J Alzheimers Dis JAD. 2018;61:1493–507. 29278888 10.3233/JAD-170540 PMC5798531

[29] Beheshti I, Demirel H, Matsuda H. Classification of Alzheimer’s disease and prediction of mild cognitive impairment-to-Alzheimer’s conversion from structural magnetic resource imaging using feature ranking and a genetic algorithm. Comput Biol Med. 2017;83:109–19. 28260614 10.1016/j.compbiomed.2017.02.011

[30] Lim BY, Lai KW, Haiskin K, Kulathilake KASH, Ong ZC, Hum YC, et al. Deep learning model for prediction of progressive mild cognitive impairment to Alzheimer’s disease using structural MRI. Front Aging Neurosci. 2022;14:876202. 35721012 10.3389/fnagi.2022.876202 PMC9201448

[31] Ocasio E, Duong TQ. Deep learning prediction of mild cognitive impairment conversion to Alzheimer’s disease at 3 years after diagnosis using longitudinal and whole-brain 3D MRI. PeerJ Comput Sci. 2021;7:e560. 34141888 10.7717/peerj-cs.560 PMC8176545

[32] Rolls ET, Huang C-C, Lin C-P, Feng J, Joliot M. Automated anatomical labelling atlas 3. Neuroimage. 2020;206:116189. 31521825 10.1016/j.neuroimage.2019.116189

[33] Eickhoff SB, Stephan KE, Mohlberg H, Grefkes C, Fink GR, Amunts K, et al. A new SPM toolbox for combining probabilistic cytoarchitectonic maps and functional imaging data. Neuroimage. 2005;25:1325–35. 15850749 10.1016/j.neuroimage.2004.12.034

[34] Eickhoff SB, Heim S, Zilles K, Amunts K. Testing anatomically specified hypotheses in functional imaging using cytoarchitectonic maps. Neuroimage. 2006;32:570–82. 16781166 10.1016/j.neuroimage.2006.04.204

[35] Eickhoff SB, Paus T, Caspers S, Grosbras M-H, Evans AC, Zilles K, et al. Assignment of functional activations to probabilistic cytoarchitectonic areas revisited. Neuroimage. 2007;36:511–21. 17499520 10.1016/j.neuroimage.2007.03.060

[36] Aisen PS, Petersen RC, Donohue M, Weiner MW, Alzheimer’s Disease Neuroimaging Initiative. Alzheimer’s Disease Neuroimaging Initiative 2 Clinical Core: Progress and plans. Alzheimers Dement. 2015;11:734–9. 26194309 10.1016/j.jalz.2015.05.005 PMC4643840

[37] Aisen PS, Petersen RC, Donohue MC, Gamst A, Raman R, Thomas RG, et al. Clinical core of the Alzheimer’s disease neuroimaging initiative: progress and plans. Alzheimers Dement. 2010;6:239–46. 20451872 10.1016/j.jalz.2010.03.006 PMC2867843

[38] Fischl B, Dale AM. Measuring the thickness of the human cerebral cortex from magnetic resonance images. Proc Natl Acad Sci. 2000;97:11050–5. 10984517 10.1073/pnas.200033797 PMC27146

[39] Ezzati A, Zammit AR, Habeck C, Hall CB, Lipton RB. Detecting biological heterogeneity patterns in ADNI amnestic mild cognitive impairment based on volumetric MRI. Brain Imaging Behav. 2020;14:1792–804. 31104279 10.1007/s11682-019-00115-6 PMC7203761

[40] Desikan RS, Ségonne F, Fischl B, Quinn BT, Dickerson BC, Blacker D, et al. An automated labeling system for subdividing the human cerebral cortex on MRI scans into gyral based regions of interest. Neuroimage. 2006;31:968–80. 16530430 10.1016/j.neuroimage.2006.01.021

[41] Fischl B, Salat DH, Busa E, Albert M, Dieterich M, Haselgrove C, et al. Whole brain segmentation. Neuron. 2002;33:341–55. 11832223 10.1016/s0896-6273(02)00569-x

[42] Fischl B, Salat DH, Van Der Kouwe AJW, Makris N, Ségonne F, Quinn BT, et al. Sequence-independent segmentation of magnetic resonance images. Neuroimage. 2004;23:S69-84. 15501102 10.1016/j.neuroimage.2004.07.016

[43] Mofrad SA, Lundervold A, Lundervold AS. A predictive framework based on brain volume trajectories enabling early detection of Alzheimer’s disease. Comput Med Imaging Graph. 2021;90:101910. 33862355 10.1016/j.compmedimag.2021.101910

[44] Han X, Jovicich J, Salat D, Van Der Kouwe A, Quinn B, Czanner S, et al. Reliability of MRI-derived measurements of human cerebral cortical thickness: the effects of field strength, scanner upgrade and manufacturer. Neuroimage. 2006;32:180–94. 16651008 10.1016/j.neuroimage.2006.02.051

[45] Palmqvist S, Mattsson N, Hansson O, for the Alzheimer’s Disease Neuroimaging Initiative. Cerebrospinal fluid analysis detects cerebral amyloid-β accumulation earlier than positron emission tomography. Brain. 2016;139:1226–36. 26936941 10.1093/brain/aww015 PMC4806222

[46] Shaw LM, Vanderstichele H, Knapik-Czajka M, Clark CM, Aisen PS, Petersen RC, et al. Cerebrospinal fluid biomarker signature in Alzheimer’s Disease Neuroimaging Initiative subjects. Ann Neurol. 2009;65:403–13. 19296504 10.1002/ana.21610 PMC2696350

[47] Hansson O, Seibyl J, Stomrud E, Zetterberg H, Trojanowski JQ, Bittner T, et al. CSF biomarkers of Alzheimer’s disease concord with amyloid-β PET and predict clinical progression: a study of fully automated immunoassays in BioFINDER and ADNI cohorts. Alzheimers Dement. 2018;14:1470–81. 29499171 10.1016/j.jalz.2018.01.010 PMC6119541

[48] Corder EH, Saunders AM, Strittmatter WJ, Schmechel DE, Gaskell PC, Small GW, et al. Gene dose of apolipoprotein E type 4 allele and the risk of Alzheimer’s disease in late onset families. Science. 1993;261:921–3. 8346443 10.1126/science.8346443

[49] Spampinato MV, Rumboldt Z, Hosker RJ, Mintzer JE, For the Alzheimer’s Disease Neuroimaging Initiative. Apolipoprotein E and Gray Matter Volume Loss in Patients with Mild Cognitive Impairment and Alzheimer Disease. Radiology. 2011;258:843–52. 21163916 10.1148/radiol.10100307 PMC6939949

[50] Gupta Y, Lama RK, Kwon G-R, For the Alzheimer’s Disease Neuroimaging Initiative. Prediction and classification of Alzheimer’s disease based on combined features from apolipoprotein-E genotype, cerebrospinal fluid, MR, and FDG-PET imaging biomarkers. Front Comput Neurosci. 2019;13:72. 31680923 10.3389/fncom.2019.00072 PMC6805777

[51] Saykin AJ, Shen L, Foroud TM, Potkin SG, Swaminathan S, Kim S, et al. Alzheimer’s Disease Neuroimaging Initiative biomarkers as quantitative phenotypes: genetics core aims, progress, and plans. Alzheimers Dement. 2010;6:265–73. 20451875 10.1016/j.jalz.2010.03.013 PMC2868595

[52] Crane PK, Carle A, Gibbons LE, Insel P, Mackin RS, Gross A, et al. Development and assessment of a composite score for memory in the Alzheimer’s Disease Neuroimaging Initiative (ADNI). Brain Imaging Behav. 2012;6:502–16. 22782295 10.1007/s11682-012-9186-z PMC3806057

[53] Gibbons LE, Carle AC, Mackin RS, Harvey D, Mukherjee S, Insel P, et al. A composite score for executive functioning, validated in Alzheimer’s Disease Neuroimaging Initiative (ADNI) participants with baseline mild cognitive impairment. Brain Imaging Behav. 2012;6:517–27. 22644789 10.1007/s11682-012-9176-1 PMC3684181

[54] Chen T, Guestrin C. XGBoost: a scalable tree boosting system. Proc 22nd ACM SIGKDD Int Conf Knowl Discov Data Min [Internet]. San Francisco California USA: ACM; 2016 [cited 2023 Apr 13]. p. 785–94. Available from: 10.1145/2939672.2939785

[55] Rossum G van, Drake FL. The Python language reference. Release 3.0.1 [Repr.]. Hampton, NH: Python Software Foundation; 2010.

[56] Pedregosa F, Varoquaux G, Gramfort A, Michel V, Thirion B, Grisel O, et al. Scikit-learn: machine learning in Python. 2012 [cited 2023 Apr 13]; Available from: https://arxiv.org/abs/1201.0490

[57] Kluyver T, Ragan-Kelley B, P&#233, Rez F, Granger B, Bussonnier M, et al. Jupyter Notebooks – a publishing format for reproducible computational workflows. Position Power Acad Publ Play Agents Agendas. 2016;87–90.

[58] Hashmi A, Osman AH. Brain tumor classification using conditional segmentation with residual network and attention approach by Extreme Gradient Boost. Appl Sci. 2022;12:10791.

[59] Kaufmann T, van der Meer D, Doan NT, Schwarz E, Lund MJ, Agartz I, et al. Common brain disorders are associated with heritable patterns of apparent aging of the brain. Nat Neurosci. 2019;22:1617–23. 31551603 10.1038/s41593-019-0471-7 PMC6823048

[60] Lange AG, Barth C, Kaufmann T, Anatürk M, Suri S, Ebmeier KP, et al. The maternal brain: region-specific patterns of brain aging are traceable decades after childbirth. Hum Brain Mapp. 2020;41:4718–29. 32767637 10.1002/hbm.25152 PMC7555081

[61] García V, Mollineda RA, Sánchez JS. Index of balanced accuracy: a performance measure for skewed class distributions. In: Araujo H, Mendonça AM, Pinho AJ, Torres MI, editors. Pattern Recognit Image Anal. Berlin, Heidelberg: Springer; 2009. p. 441–8.

[62] Moreno-Ibarra M-A, Villuendas-Rey Y, Lytras MD, Yáñez-Márquez C, Salgado-Ramírez J-C. Classification of diseases using machine learning algorithms: a comparative study. Mathematics. 2021;9:1817.

[63] Mosca E, Szigeti F, Tragianni S, Gallagher D, Groh G. SHAP-based explanation methods: a review for NLP interpretability. Proc 29th Int Conf Comput Linguist [Internet]. Gyeongju, Republic of Korea: International Committee on Computational Linguistics; 2022 [cited 2023 Apr 13]. p. 4593–603. Available from: https://aclanthology.org/2022.coling-1.406

[64] Hicks SA, Strümke I, Thambawita V, Hammou M, Riegler MA, Halvorsen P, et al. On evaluation metrics for medical applications of artificial intelligence. Sci Rep. 2022;12:5979. 35395867 10.1038/s41598-022-09954-8 PMC8993826

[65] Goutte C, Gaussier E. A probabilistic interpretation of precision, recall and F-score, with implication for evaluation. In: Losada DE, Fernández-Luna JM, editors. Adv Inf Retr [Internet]. Berlin, Heidelberg: Springer Berlin Heidelberg; 2005 [cited 2023 Oct 10]. p. 345–59. Available from: 10.1007/978-3-540-31865-1_25

[66] M H, M S. A review on evaluation metrics for data classification evaluations. Int J Data Min Knowl Manag Process. 2015;5:01–11.

[67] Alzheimer’s disease facts and figures. Alzheimers Dement. 2023;19:1598–695. 36918389 10.1002/alz.13016

[68] Xie L, Das SR, Wisse LEM, Ittyerah R, De Flores R, Shaw LM, et al. Baseline structural MRI and plasma biomarkers predict longitudinal structural atrophy and cognitive decline in early Alzheimer’s disease. Alzheimers Res Ther. 2023;15:79. 37041649 10.1186/s13195-023-01210-z PMC10088234

[69] Vemuri P, Wiste HJ, Weigand SD, Shaw LM, Trojanowski JQ, Weiner MW, et al. MRI and CSF biomarkers in normal, MCI, and AD subjects: predicting future clinical change. Neurology. 2009;73:294–301. 19636049 10.1212/WNL.0b013e3181af79fb PMC2715214

[70] Schaeverbeke J, Delmotte K, Vandenberghe R, Poesen K. Prognostic value of amyloid/tau/neurodegeneration (ATN) classification based on diagnostic cerebrospinal fluid samples for Alzheimer’s disease. Alzheimers Dement [Internet]. 2021 [cited 2023 Aug 18];17. Available from: 10.1002/alz.051036 10.1186/s13195-021-00817-4 PMC8059197 33879243

[71] Maruszak A, Thuret S. Why looking at the whole hippocampus is not enoughâ€”a critical role for anteroposterior axis, subfield and activation analyses to enhance predictive value of hippocampal changes for Alzheimerâ€TMs disease diagnosis. Front Cell Neurosci [Internet]. 2014 [cited 2023 Aug 18];8. Available from: 10.3389/fncel.2014.00095/abstract 10.3389/fncel.2014.00095 PMC3978283 24744700

[72] Kulason S, Xu E, Tward DJ, Bakker A, Albert M, Younes L, et al. Entorhinal and transentorhinal atrophy in preclinical Alzheimer’s disease. Front Neurosci. 2020;14:804. 32973425 10.3389/fnins.2020.00804 PMC7472871

[73] Rombouts SARB, Barkhof F, Witter MP, Scheltens P. Unbiased whole-brain analysis of gray matter loss in Alzheimer’s disease. Neurosci Lett. 2000;285:231–3. 10806328 10.1016/s0304-3940(00)01067-3

[74] Baron JC, Chételat G, Desgranges B, Perchey G, Landeau B, De La Sayette V, et al. In vivo mapping of gray matter loss with voxel-based morphometry in mild Alzheimer’s disease. Neuroimage. 2001;14:298–309. 11467904 10.1006/nimg.2001.0848

[75] Hirao K, Ohnishi T, Matsuda H, Nemoto K, Hirata Y, Yamashita F, et al. Functional interactions between entorhinal cortex and posterior cingulate cortex at the very early stage of Alzheimer’s disease using brain perfusion single-photon emission computed tomography. Nucl Med Commun. 2006;27:151–6. 16404228 10.1097/01.mnm.0000189783.39411.ef

[76] Ishii K, Sasaki H, Kono AK, Miyamoto N, Fukuda T, Mori E. Comparison of gray matter and metabolic reduction in mild Alzheimer’s disease using FDG-PET and voxel-based morphometric MR studies. Eur J Nucl Med Mol Imaging. 2005;32:959–63. 15800784 10.1007/s00259-004-1740-5

[77] Di Paola M, Macaluso E, Carlesimo GA, Tomaiuolo F, Worsley KJ, Fadda L, et al. Episodic memory impairment in patients with Alzheimer’s disease is correlated with entorhinal cortex atrophy A voxel-based morphometry study. J Neurol. 2007;254:774–81. 17404777 10.1007/s00415-006-0435-1

[78] Frisoni GB. Structural correlates of early and late onset Alzheimer’s disease: voxel based morphometric study. J Neurol Neurosurg Psychiatry. 2005;76:112–4. 15608008 10.1136/jnnp.2003.029876 PMC1739332

[79] Shiino A, Watanabe T, Maeda K, Kotani E, Akiguchi I, Matsuda M. Four subgroups of Alzheimer’s disease based on patterns of atrophy using VBM and a unique pattern for early-onset disease. Neuroimage. 2006;33:17–26. 16904912 10.1016/j.neuroimage.2006.06.010

[80] Whitwell JL. Voxel-based morphometry: an automated technique for assessing structural changes in the brain. J Neurosci. 2009;29:9661–4. 19657018 10.1523/JNEUROSCI.2160-09.2009 PMC6666603

[81] Mieling M, Meier H, Bunzeck N. Structural degeneration of the Nucleus basalis of Meynert in mild cognitive impairment and Alzheimer’s disease – evidence from an MRI-based meta-analysis. Neurosci Biobehav Rev. 2023;105393. 10.1016/j.neubiorev.2023.105393 37717861

[82] Mofrad SA, Lundervold AJ, Vik A, Lundervold AS. Cognitive and MRI trajectories for prediction of Alzheimer’s disease. Sci Rep. 2021;11:2122. 33483535 10.1038/s41598-020-78095-7 PMC7822915

[83] Albert M, Zhu Y, Moghekar A, Mori S, Miller MI, Soldan A, et al. Predicting progression from normal cognition to mild cognitive impairment for individuals at 5 years. Brain. 2018;141:877–87. 29365053 10.1093/brain/awx365 PMC5837651

[84] Cheng CPW, Cheng ST, Tam CWC, Chan WC, Chu WCW, Lam LCW. Relationship between cortical thickness and neuropsychological performance in normal older adults and those with mild cognitive impairment. Aging Dis. 2018;9:1020. 30574415 10.14336/AD.2018.0125 PMC6284757

[85] Singh V, Chertkow H, Lerch JP, Evans AC, Dorr AE, Kabani NJ. Spatial patterns of cortical thinning in mild cognitive impairment and Alzheimer’s disease. Brain. 2006;129:2885–93. 17008332 10.1093/brain/awl256

[86] Julkunen V, Niskanen E, Koikkalainen J, Herukka S-K, Pihlajamäki M, Hallikainen M, et al. Differences in cortical thickness in healthy controls, subjects with mild cognitive impairment, and Alzheimer’s disease patients: a longitudinal study. J Alzheimers Dis. 2010;21:1141–51. 21504134 10.3233/jad-2010-100114

[87] Núñez C, Callén A, Lombardini F, Compta Y, Stephan-Otto C, Alzheimer’s Disease Neuroimaging Initiative. Different cortical gyrification patterns in Alzheimer’s disease and impact on memory performance. Ann Neurol. 2020;88:67–80. 32277502 10.1002/ana.25741

[88] Bonthius DJ, Solodkin A, Van Hoesen GW. Pathology of the insular cortex in Alzheimer disease depends on cortical architecture. J Neuropathol Exp Neurol. 2005;64:910–22. 16215463 10.1097/01.jnen.0000182983.87106.d1

[89] Christen-Zaech S, Kraftsik R, Pillevuit O, Kiraly M, Martins R, Khalili K, et al. Early olfactory involvement in Alzheimer’s disease. Can J Neurol Sci J Can Sci Neurol. 2003;30:20–5. 10.1017/s0317167100002389 12619779

[90] Augustine J. Circuitry and functional aspects of the insular lobe in primates including humans. Brain Res Rev. 1996;22:229–44. 8957561 10.1016/s0165-0173(96)00011-2

[91] Moon Y, Moon W-J, Kim H, Han S-H. Regional atrophy of the insular cortex is associated with neuropsychiatric symptoms in Alzheimer’s disease patients. Eur Neurol. 2014;71:223–9. 24480815 10.1159/000356343

[92] Fotuhi M, Do D, Jack C. Modifiable factors that alter the size of the hippocampus with ageing. Nat Rev Neurol. 2012;8:189–202. 22410582 10.1038/nrneurol.2012.27

[93] Freitas S, Simões MR, Alves L, Santana I. Montreal cognitive assessment: validation study for mild cognitive impairment and Alzheimer disease. Alzheimer Dis Assoc Disord. 2013;27:37–43. 22193353 10.1097/WAD.0b013e3182420bfe

[94] Yue L, Hu D, Zhang H, Wen J, Wu Y, Li W, et al. Prediction of 7-year’s conversion from subjective cognitive decline to mild cognitive impairment. Hum Brain Mapp. 2021;42:192–203. 33030795 10.1002/hbm.25216 PMC7721238

[95] Karaman BK, Mormino EC, Sabuncu MR. Machine learning based multi-modal prediction of future decline toward Alzheimer’s disease: an empirical study. PLOS ONE. 2022;17:e0277322. 36383528 10.1371/journal.pone.0277322 PMC9668188

[96] Muhammed Niyas KP, Thiyagarajan P. A systematic review on early prediction of Mild cognitive impairment to alzheimers using machine learning algorithms. Int J Intell Netw. 2023;4:74–88.

[97] Moscovitch M, Cabeza R, Winocur G, Nadel L. Episodic memory and beyond: the hippocampus and neocortex in transformation. Annu Rev Psychol. 2016;67:105–34. 26726963 10.1146/annurev-psych-113011-143733 PMC5060006

[98] Schultz H, Sommer T, Peters J. The role of the human entorhinal cortex in a representational account of memory. Front Hum Neurosci [Internet]. 2015 [cited 2023 Sep 22];9. 10.3389/fnhum.2015.00628/abstract 10.3389/fnhum.2015.00628 PMC4653609 26635581

[99] Braak H, Braak E. Neuropathological stageing of Alzheimer-related changes. Acta Neuropathol (Berl). 1991;82:239–59. 1759558 10.1007/BF00308809

[100] Karas GB, Scheltens P, Rombouts SARB, Visser PJ, Van Schijndel RA, Fox NC, et al. Global and local gray matter loss in mild cognitive impairment and Alzheimer’s disease. Neuroimage. 2004;23:708–16. 15488420 10.1016/j.neuroimage.2004.07.006

[101] Gerardin E, Chételat G, Chupin M, Cuingnet R, Desgranges B, Kim H-S, et al. Multidimensional classification of hippocampal shape features discriminates Alzheimer’s disease and mild cognitive impairment from normal aging. Neuroimage. 2009;47:1476–86. 19463957 10.1016/j.neuroimage.2009.05.036 PMC3001345

[102] Hett K, Ta V-T, Catheline G, Tourdias T, Manjón JV, Coupé P, et al. Multimodal hippocampal subfield grading for Alzheimer’s disease classification. Sci Rep. 2019;9:13845. 31554909 10.1038/s41598-019-49970-9 PMC6761169

[103] Frisoni GB, Fox NC, Jack CR, Scheltens P, Thompson PM. The clinical use of structural MRI in Alzheimer disease. Nat Rev Neurol. 2010;6:67–77. 20139996 10.1038/nrneurol.2009.215 PMC2938772

[104] Arrondo P, Elía-Zudaire Ó, Martí-Andrés G, Fernández-Seara MA, Riverol M. Grey matter changes on brain MRI in subjective cognitive decline: a systematic review. Alzheimers Res Ther. 2022;14:98. 35869559 10.1186/s13195-022-01031-6 PMC9306106

[105] Zeng Q, Qiu T, Li K, Luo X, Wang S, Xu X, et al. Increased functional connectivity between nucleus basalis of Meynert and amygdala in cognitively intact elderly along the Alzheimer’s continuum. NeuroImage Clin. 2022;36:103256. 36451361 10.1016/j.nicl.2022.103256 PMC9668640

[106] Zhang T, Liao Q, Zhang D, Zhang C, Yan J, Ngetich R, et al. Predicting MCI to AD conversation using integrated sMRI and rs-fMRI: machine learning and graph theory approach. Front Aging Neurosci. 2021;13:688926. 34421570 10.3389/fnagi.2021.688926 PMC8375594

[107] Suk H-I, Lee S-W, Shen D. Latent feature representation with stacked auto-encoder for AD/MCI diagnosis. Brain Struct Funct. 2015;220:841–59. 24363140 10.1007/s00429-013-0687-3 PMC4065852

[108] Cabeza R, Dolcos F, Graham R, Nyberg L. Similarities and differences in the neural correlates of episodic memory retrieval and working memory. Neuroimage. 2002;16:317–30. 12030819 10.1006/nimg.2002.1063

[109] Makino Y, Yokosawa K, Takeda Y, Kumada T. Visual search and memory search engage extensive overlapping cerebral cortices: an fMRI study. Neuroimage. 2004;23:525–33. 15488401 10.1016/j.neuroimage.2004.06.026

[110] Martínez-Florez JF, Osorio JD, Cediel JC, Rivas JC, Granados-Sánchez AM, López-Peláez J, et al. Short-term memory binding distinguishing amnestic mild cognitive impairment from healthy aging: a machine learning study. J Alzheimers Dis. 2021;81:729–42. 33814438 10.3233/JAD-201447

[111] Zhang X-D. Machine learning. Matrix algebra approach Artif Intell [Internet]. Singapore: Springer Singapore; 2020 [cited 2024 Mar 7]. p. 223–440. Available from: 10.1007/978-981-15-2770-8_6

[112] Herm L-V, Heinrich K, Wanner J, Janiesch C. Stop ordering machine learning algorithms by their explainability! A user-centered investigation of performance and explainability. Int J Inf Manag. 2023;69:102538.

[113] Mieling M, Göttlich M, Yousuf M, Bunzeck N. Basal forebrain activity predicts functional degeneration in the entorhinal cortex in Alzheimer’s disease. Brain Commun. 2023;5:fcad262. 37901036 10.1093/braincomms/fcad262 PMC10608112

[114] Feng F, Huang W, Meng Q, Hao W, Yao H, Zhou B, et al. Altered volume and structural connectivity of the hippocampus in Alzheimer’s disease and amnestic mild cognitive impairment. Front Aging Neurosci. 2021;13:705030. 34675796 10.3389/fnagi.2021.705030 PMC8524052

[115] Vieira S, Pinaya WHL, Mechelli A. Using deep learning to investigate the neuroimaging correlates of psychiatric and neurological disorders: methods and applications. Neurosci Biobehav Rev. 2017;74:58–75. 28087243 10.1016/j.neubiorev.2017.01.002

