# Supplementary material for: Predicting the progression of MCI and Alzheimer’s disease on structural brain integrity and other features with machine learning
Source: GeroScience. 2025 Apr 26;48(1):463–87. doi: 10.1007/s11357-025-01626-5 (PMC12972442; doi:10.1007/s11357-025-01626-5)
Supplement: Supplementary file 1 — Supplementary file1 (DOCX 43.2 KB) [file 11357_2025_1626_MOESM1_ESM.docx]

**Supplementary material**

***Predicting the progression of MCI and Alzheimer’s disease***

***on structural brain integrity and other features with machine learning***

**^*^**Marthe Mieling ^1^, **^*^**Mushfa Yousuf ^1^, Nico Bunzeck ^1, 2^, ^**^for the Alzheimer's Disease Neuroimaging Initiative

^1^ Department of Psychology, University of Lübeck, 23562 Lübeck, Germany

^2^ Center of Brain, Behavior and Metabolism (CBBM), University of Lübeck, Ratzeburger Allee 160, 23562 Lübeck, Germany

Corresponding authors:

Marthe Mieling, Nico Bunzeck

Department of Psychology, University of Lübeck, Ratzeburger Allee 160, 23562 Lübeck, Germany

Phone: +49-(0) 451 3101 3635

E-Mail: [m.mieling@uni-luebeck.de](mailto:m.mieling@uni-luebeck.de), [nico.bunzeck@uni-luebeck.de](mailto:nico.bunzeck@uni-luebeck.de)

* Equal contributions

**Table S1.** Demographics, information on APOE4 genotype and harmonized CSF assays.

| **Analysis 1** | **CN** | **MCI** | **AD** | **Test - χ²/ Fisher’s F** |
| --- | --- | --- | --- | --- |
| n (total)=568  ADNI-GO/2 (n=334) / 3 (n=234) | 271  128/142 | 193  132/61 | 104  73/31 | χ²=73.8, p<.001***  χ²=94.1, p<.001*** |
| Manufacturer  Philips (n=95) / Siemens (n=362) / GE (n=111) | 36/184/51 | 33/123/37 | 26/55/23 | χ²=9.29, p=0.054 |
| FreeSurfer versions (5.1, 6.0) | 129/142 | 132/61 | 73/31 | χ²=26.9, p<.001*** |
| Age | 71.0 (5.99) | 70.8 (7.47) | 73.9 (8.24) | F(2,565)=7.89, p<.001*** |
| Female (n=288) / Male (n=280) | 164/107 | 89/104 | 35/69 | χ²=24.2, p<.001*** |
| Education (in years) | 16.7 (2.28) | 16.1 (2.72) | 15.9 (2.59) | F(2,565)=5.77,  p=0.003** |
| APOE 4 (0/1/2) | 182/82/7 | 123/53/17 | 33/47/24 | χ²=61.3 p<.001*** |
| Aβ | 1415 (656) | 1181 (582) | 664 (394) | F(2,565)= 60.8, p<.001*** |
| pTau | 20.4 (8.52) | 24.5 (13.1) | 36.8 (14.4) | F(2,565)=76.4, p<.001*** |
| pTau/Amyloid ratio | 0.0184 (0.0147) | 0.0269 (0.0221) | 0.0661 (0.0361) | F(2,565)=171, p<.001*** |
| **Analysis 2** | **CN stable** | **CN-to-MCI converter** | **Test - χ²/ t** | |
| n (total)=92  ADNI1 (n=30) / ADNI-GO/2 (n=38) / 3 (n=24) | 46  15/19/12 | 46  15/19/12 | χ²=0.00, p=1.0  χ²=90.00, p=1.0 | |
| Manufacturer  Philips (n=6) / Siemens (n=20) / GE (n=20) | 6/20/20 | 8/19/19 | χ²=0.337, p=0.845 | |
| Scanner fieldstrength (1.5 / 3.0) | 25/21 | 25/21 | χ²=0.00, p=1.0 | |
| FreeSurfer versions (4.3, 5.1, 6.0) | 25/9/12 | 25/9/12 | χ²=0.00, p=1.0 | |
| Age | 73.6 (6.37) | 77.9 (5.94) | t=3.34, p<.001*** | |
| Female (n=42) / Male (n=50) | 23/23 | 19/27 | χ²=0.701, p=0.402 | |
| Education (in years) | 16.5 (2.61) | 16.3 (2.61) | t=-0.28, p=0.78 | |
| APOE 4 (0/1/2) | 29/15/2 | 28/15/3 | χ²=0.218 p=0.897 | |
| Aβ if available (n=32) | 1129.55 (465.18) | 969.69 (680.58) | t=-0.776, p=0.444 | |
| pTau if available (n=32) | 20.61 (6.72) | 25.96 (8.11) | t=2.03, p=0.051 | |
| pTau/Amyloid ratio if available (n=32) | 0.0231 (0.0372) | 0.0372 (0.0224) | t=2.122, p=0.042* | |
| Stable time for non-converters; time until next follow-up for converters (in months) | 70.54 (47.94) | 15.0 (8.35) | t=-7.75, p<.001*** | |
| **Analysis 3** | **MCI stable** | **MCI-to-AD converters** | **Test - χ²/ t** | |
| n (total)=378  ADNI1 (n=228) / ADNI-GO/2 (n=113) / 3 (n=37) | 189  112/58/19 | 189  116/55/18 | χ²=0.00, p=1.0  χ²=0.177, p=0.915 | |
| Manufacturer  Philips (n=57) / Siemens (n=180) / GE (n=141) | 32/91/66 | 25/89/75 | χ²=1.46, p=0.483 | |
| Scanner field strength (1.5 / 3.0) | 111/78 | 125/64 | χ²=2.21, p=0.137 | |
| FreeSurfer versions (4.3, 5.1, 6.0) | 111/59/19 | 125/46/18 | χ²=0.00, p=0.291 | |
| Age | 73.6 (7.44) | 74.4 (6.89) | t=1.09, p=0.276 | |
| Female (n=155) / Male (n=223) | 77/112 | 78/111 | χ²=0.011, p=0.917 | |
| Education (in years) | 15.6 (3.05) | 15.9 (2.88) | t=0.935, p=0.35 | |
| APOE 4 (0/1/2) | 101/68/20 | 52/109/28 | χ²=26.5 p<.001*** | |
| Aβ if available (n=88) | 1145 (572) | 719 (339) | t=-4.25, p<.001*** | |
| pTau if available (n=88) | 25.1 (12.7) | 35.1 (15.9) | t=3.27, p=0.002** | |
| pTau/Amyloid ratio if available (n=88) | 0.0307 (0.0256) | 0.0576 (0.0291) | t=4.59, p<.001*** | |
| Stable for non-converters; time until next follow-up for converters (in months) | 52.5 (93.5) | 9.53 (5.85) | t=-6.3, p<.001*** | |

Information of the final sample from analysis 1, 2 and 3 grouped by diagnoses. Means and standard deviation (SD) are represented and the respective t-test or chi-square test to investigate possible group differences. Baseline clinical diagnosis: CN=cognitive normal; MCI=mild cognitive impairment; AD=Alzheimer’s Disease dementia. Age and education were assessed in years. APOE4 status: no allel / 1 allel / 2 allels. Aβ=amyloid-β in pg/ml as concentration of the amyloid- β 1-42 peptide. pTau=in pg/ml as CSF concentration of hyperphosphorylated tau. *p<0.05, **p<0.01, ***p<0.001

**Table S2.** Neuropsychological test results at the first measurement, for analysis 1 CN vs. MCI vs. AD, analysis 2 CN-stable vs. CN-converter and analysis 3 MCI-stable vs. MCI-converter. Shown are mean values and numbers in brackets represent one SD.

| **Neuropsychological test** | **mean values (SD)** | | | **F- value** | **P- value** |
| --- | --- | --- | --- | --- | --- |
| **Analysis 1** | **CN MCI AD** | | |  |  |
| MEM score  EF score  MMSE  ADAS-Cog 13  CDRSB  MoCA  Clock drawing | 1.13 (0.57)  1.16 (0.81)  29.2(1.05)  8.01 (4.07)^2^  0.02 (0.12)  26.4 (2.34)^6^  4.7 (0.59)^1^ | 0.487 (0.61)  0.538 (0.87)  28.2 (1.64)^1^  13.3(5.56)  1.36 (0.88)  23.8(2.87)  4.48 (0.8) | -0.88 (0.5)  -0.78(1.02)^1^  23.1(1.96)  30.7(7.78)^3^  4.59 (1.63)  17.5 (4.29)^5^  3.42 (1.48) | F(2,562)=410.4  F(2,561)=161.96  F(2,561)=591.29  F(2,557)=583.24  F(2,562)=961.92  F(2,551)=297.01  F(2,561)=70.971 | <0.001*  <0.001*  <0.001*  <0.001*  <0.001*  <0.001*  <0.001* |
| **Analysis 2** | **CN stable** | **CN converters** | | F(1,87)=9.88  F(1,87)=2.76  F(1,87)=0.1  F(1,87)=14.44  F(1,87)=7.24  F(1,87)=7.31  F(1,87)=2.1 | =0.002*  =0.1  =0.75  <0.001*  =0.009  =0.009  =0.151 |
| MEM score  EF score  MMSE  ADAS-Cog 13  CDRSB  MoCA  Clock drawing | 1.09 (0.54)  0.91 (0.84)  29 (1.19)  7.91 (3.73)  0.01 (0.07)  26.2 (2.29)^15^  4.74 (0.49) | 0.6 (0.58)  0.53 (0.67)  28.8 (1.4)  12.5 (5.42)  0.174 (0.41)  24.3 (2.52)^16^  4.59 (0.65) | |  |  |
| **Analysis 3** | **MCI stable** | **MCI converters** | | F(1,371)=142.71  F(1,371)=38.42  F(1,371)=45.32  F(1,371)=136.59  F(1,372)=135.05  F(1,142)=32.926  F(1,372)=15.04 | <0.001*  <0.001*  <0.001*  <0.001*  <0.001*  <0.001*  <0.001* |
| MEM score  EF score  MMSE  ADAS-Cog 13  CDRSB  MoCA  Clock drawing | 0.229(0.66)  0.205 (0.85)  27.4 (2.21)^1^  15.7 (6.5)^1^  1.43 (0.8)^1^  23.08 (3.25)^113^  4.39 (0.8) | -0.478 (0.52)^1^  -0.314 (0.831)^2^  25.8(2.4)^1^  23.5 (6.59)^1^  2.57 (1.06)  20.8 (3.18)^118^  4.02 (1.03)^1^ | |  |  |

The mean values with standard deviation (SD) for cognitive normal (CN); Mild cognitive impairment (MCI) and Alzheimer’s disease dementia (AD). The upper numbers represent the missing values that were excluded. The AD groups showed worse performance in all neuropsychological tests followed by MCI and CN. MEM: memory function score; EF: executive function score; MMSE: Mini-Mental State Examination; ADAS-Cog 13: Alzheimer's Disease Assessment Scale- Cognition Subscale, 13 tasks; CDRSB: Clinical Dementia Rating Scale; MoCA: Montreal-Cognitive-Assessment; Clock drawing: clock drawing test

* significant after Bonferroni correction p<0.05/n (n=7 tests).

**Table S3.** Included brain regions with volume and thickness for all analyses based on the cortical and subcortical parcellation

| **Regions with volume** | **Regions with thickness** |
| --- | --- |
| \| Pallidum_volume \| \| --- \| \| Paracentral_volume \| \| Parahippocampal_volume \| \| ParsOpercularis_volume \| \| ParsOrbitalis_volume \| \| ParsTriangularis_volume \| \| Pericalcarine_volume \| \| Postcentral_volume \| \| PosteriorCingulate_volume \| \| Precentral_volume \| \| Precuneus_volume \| \| Putamen_volume \| \| RostralAnteriorCingulate_volume \| \| RostralMiddleFrontal_volume \| \| SuperiorFrontal_volume \| \| SuperiorParietal_volume \| \| SuperiorTemporal_volume \| \| Supramarginal_volume \| \| TemporalPole_volume \| \| Thalamus_volume \| \| TransverseTemporal_volume \| \| VentralDC_volume \| \| Insula_volume \| \| Amygdala_volume \| \| Bankssts_volume \| \| CaudalAnteriorCingulate_volume \| \| CaudalMiddleFrontal_volume \| \| Caudate_volume \| \| CerebellumCortex_volume \| \| CerebellumWM_volume \| \| ChoroidPlexus_volume \| \| Cuneus_volume \| \| Entorhinal_volume \| \| FrontalPole_volume \| \| Fusiform_volume \| \| Hippocampus_volume \| \| InferiorParietal_volume \| \| InferiorTemporal_volume \| \| IsthmusCingulate_volume \| \| LateralOccipital_volume \| \| LateralOrbitofrontal_volume \| \| Lingual_volume \| \| MedialOrbitofrontal_volume \| \| MiddleTemporal_volume \| \| AccumbensArea_volume \| \| Brainstem_volume \| | \| Paracentral_thickness \| \| --- \| \| Parahippocampal_thickness \| \| Opercularis_thickness \| \| ParsOrbitalis_thickness \| \| ParsTriangularis_thickness \| \| Pericalcarine_thickness \| \| Postcentral_thickness \| \| PosteriorCingulate_thickness \| \| Precentral_thickness \| \| Precuneus_thickness \| \| RostralAnteriorCingulate_thickness \| \| RostralMiddleFrontal_thickness \| \| SuperiorFrontal_thickness \| \| SuperiorParietal_thickness \| \| SuperiorTemporal_thickness \| \| Supramarginal_thickness \| \| TemporalPole_thickness \| \| TransverseTemporal_thickness \| \| Insula_thickness \| \| Bankssts_thickness \| \| CaudalAnteriorCingulate_thickness \| \| CaudalMiddleFrontal_thickness \| \| Cuneus_thickness \| \| Entorhinal_thickness \| \| FrontalPole_thickness \| \| Fusiform_thickness \| \| InferiorParietal_thickness \| \| InferiorTemporal_thickness \| \| IsthmusCingulate_thickness \| \| LateralOccipital_thickness \| \| LateralOrbitofrontal_thickness \| \| Lingual_thickness \| \| MedialOrbitofrontal_thickness \| \| MiddleTemporal_thickness \| |

**Table S4.** Post-hoc t-test on the entire sample for all analyses.

| **Analysis 1 (n=568)** | |  |  | | |  |
| --- | --- | --- | --- | --- | --- | --- |
| **Features** | | **CN vs. MCI** | | **CN vs. AD** | **MCI vs. AD** | |
| CSF | | U=1898, p<0.001* | | U=2148, p<0.001* | U=2772, p<0.001* | |
| EC thickness | | U=2008, p=0.001* | | U=1846, p<0.001* | U=3494, p<0.001* | |
| Hippocampus volume | | t^1^(329)=-6.66, p<0.001* | | t(373)=-20.1, p<0.001* | | t^1^(257)=-10.9, p<0.001* |
| **Analysis 2 (n=92)**  **Features** | | **CN stable vs**  **CN-to-MCI converters** | | |  |  |
| Hippocampus volume  Insula thickness  STG thickness | | t(90)=-4.69, p<0.001*  t^1^(81.9)=-3.37, p=0.001*  t(90)=-3.03, p=0.003* | | |  |  |
| **Analysis 3 with CSF (n=88)**  **Features** | **MCI stable vs**  **MCI-to-AD converters** | | | |  |  |
| Amygdala volume  CSF  EC thickness | | U=638, p=0.006*  U=451, p<0.001*  t(86)=-4.16, p<0.001* | | |  |  |
| **Analysis 3 without CSF (n=378)**  **Features** | | **MCI stable vs**  **MCI-to-AD converters** | | |  |  |
| Amygdala volume  EC thickness  Cunes volume | | U=10263 p<0.001*  U=9326, p<0.001*  U=177571.03, p=0.923 | | |  |  |

Test statistics for all post-hoc t-tests across the three analyses based on the entire samples. If the assumption of normality was violated (Shapiro-Wilk test, p<0.05), a Mann-Whitney U test was conducted. If variances were unequal (Levene’s test, p<0.05), Welch’s t-statistic is reported. If both assumptions were violated, a Mann-Whitney U test was used. EC: entorhinal cortex
¹ Welch-corrected t-test due to unequal variances

* Significant after Bonferroni correction: for analysis 1, p<0.05/n (n = 9 tests); for analyses 2 and 3, p<0.05/n (n = 3 tests).
